# Mental Imagery of the Self in Body Dysmorphic Disorder: A Mixed‐Methods Systematic Review

**DOI:** 10.1002/cpp.70229

**Published:** 2026-02-15

**Authors:** Sean Hill, Matthew Hotton, Martha Wallace, David Veale, Alex Lau‐Zhu

**Affiliations:** ^1^ Department of Experimental Psychology University of Oxford Oxford UK; ^2^ Oxford Health NHS Foundation Trust Oxford UK; ^3^ Oxford University Hospitals NHS Foundation Trust Oxford UK; ^4^ Department of Psychology, Institute of Psychiatry, Psychology and Neuroscience King's College London London UK; ^5^ Centre for Anxiety Disorders and Trauma South London and Maudsley NHS Foundation Trust London UK; ^6^ Division of Psychiatry, Department of Brain Sciences Imperial College London London UK; ^7^ Linacre College University of Oxford Oxford UK

**Keywords:** body dissatisfaction, body dysmorphic disorder, CBT, EMDR, imagery rescripting, intrusive imagery, mental imagery

## Abstract

Mental imagery has been identified as a key feature of the onset, maintenance and treatment of psychological disorders. Research on the role of mental imagery in body dysmorphic disorder (BDD), a condition hallmarked by negative sensory appraisals of the self, has been increasingly recognised in theoretical perspectives and psychological interventions. However, the scope and implications of this work have not yet been reviewed. This systematic review sought to identify the characteristics and proposed mechanisms of imagery in BDD, synthesising qualitative and quantitative data using Meta‐Integration. Quality was assessed using the Mixed Methods Appraisal Tool. Thirty‐seven studies were identified among 33 publications. Study quality was mixed with significant methodological heterogeneity. Mental imagery in BDD is consistently reported to be vivid, emotionally intense, recurrent and important in the maintenance and potentially the onset of BDD. These findings concur with theoretical frameworks of BDD (and other related conditions) which highlight the causal role of imagery and encourage the use of imagery‐based interventions. Crucial areas for future work include stronger causal tests, unpacking mechanisms, attention to individual differences and intersectionality and exploring the potential for imagery‐based approaches for innovations in treatment and prevention across the lifespan, particularly in adolescence when BDD first develops.

## Summary

- Mental imagery of the self in BDD is common, recurrent, intrusive, vivid and often depicts clients' body areas of concern in an exaggerated third‐person manner or replays distressing memories linked to negative beliefs.
- These images can be triggered by everyday situations, and individuals with BDD may engage in complex strategies to avoid or disengage from the perceived threat imposed by imagery.
- Mental imagery may, in this way, be important in the maintenance and development of BDD, with clear implications for intervention efforts.
- Practitioners should consider assessing the content and experience of imagery in clients with BDD, which can serve as opportune entry‐points for deepening formulation throughout therapy.
- Novel applications of imagery work within evidence‐based therapies are emerging and appear both feasible and clinically relevant.

## 1 Introduction

### 1.1 Body Dysmorphic Disorder (BDD)

BDD is defined in the International Classification of Diseases‐11 (ICD‐11; World Health Organisation 2019) and Diagnostic and Statistical Manual of Mental Disorders, Fifth Edition (DSM‐5; American Psychological Association 2013) as a condition characterised by intense and persistent preoccupation with one or more perceived flaws or defects in physical appearance. These perceived imperfections often appear minor, insignificant or unobservable to others, consume substantial time and mental resources, leading to significant distress and dysfunction in various areas of life. Prevalence is approximately equal across genders, with onset typically in adolescence, in line with most mental health problems (Solmi et al. 2021), persisting through adulthood with low rates of spontaneous remission (Phillips 2009, 2017).

While the content of preoccupations in BDD varies between individuals, the clinical features of behaviours, distress and functional impairments tend to follow a consistent pattern. These include: (1) the ‘perceived flaws’ (Veale 2004) related to the specific body areas of concern, most commonly skin and facial features, especially nose and hair (Phillips 2009); (2) rigidly‐held negative appraisals regarding perceived flaws, including judgements related to social unacceptability, rejection (e.g., ‘They think my teeth are yellow, so others will reject me’), perceived inability to meet desired goals (e.g., ‘I am unattractive and only attractive people can be successful’) or physical health (e.g., ‘My skin looks worse because I am getting weaker’) (Didie et al. 2010); (3) behaviours intended to alleviate distress, which may involve hiding, covering or masking the perceived flaw (e.g., make‐up, grooming and avoiding social situations), attempts to alter or remove the perceived flaw (e.g., skin‐picking, self‐surgery and formal cosmetic surgery) or hypervigilance (e.g., mirror‐checking, reassurance seeking and comparison to others). While these behaviours can offer short‐term relief, they also reinforce beliefs that their perceived flaws are significant and perpetuate cycles of distress (Veale 2004).

Importantly, body‐image psychopathology is conceptualised within this framework as existing on a continuum, with BDD at the clinical end and body dissatisfaction in community samples representing subclinical expressions (Phillips 2009). While both manifestations share substantial phenomenological and mechanistic overlap, including negative appearance evaluations, heightened self‐focused attention, shame and recurrent attempts to monitor or alter appearance (Mills et al. 2022), BDD is characterised by markedly greater levels of preoccupation and functional impairment.

The United Kingdom (UK) National Institute for Health and Care Excellence (NICE) guidelines endorse cognitive‐behavioural therapy (CBT) as a first‐line psychological intervention for BDD (NICE 2005). Recent meta‐analytic findings support the efficacy of CBT for reducing BDD symptom severity (Zhao et al. 2024), reducing scores on the BDD‐Yale Brown Obsessive Compulsive Scale (BDD‐YBOCS) significantly compared to control groups (SMD = −1.73, 95% CI = [−2.90;−0.57], *p* < 0.01) with large effect sizes after 12 weeks of treatment (SMD = −1.51, 95% CI = [−2.81;−0.21]). Despite these promising findings, previous reviews suggest only 40%–54% of patients achieve responder status, defined as a clinically significant improvement of at least 30% on the BDD‐YBOCS (Harrison et al. 2016), and only 20% achieve full remission within four years (Phillips et al. 2013). This response rate is substantially lower than for similar disorders, such as obsessive compulsive disorder (OCD), where rates typically range between 60%–80% (Mataix‐Cols et al. 2016). Benefits of CBT for BDD also appear to diminish at follow‐up beyond three months compared to control groups (SMD = −2.30, 95% CI = [−4.98, 0.37]; Zhao et al. 2024). Thus, while CBT can be an effective intervention, sustained improvements may require additional or modified therapeutic approaches.

Effective treatment adaptations for specific groups also remain a limitation of current practice (Hogg et al. 2023), including in adolescence when BDD tends to first develop (Wilhelm et al. 2014). To improve outcomes for BDD, a better understanding is needed of the mechanisms involved in the development, maintenance and amelioration of BDD (Liu et al. 2024). Here we focus on mental imagery, a key psychological process across anxiety‐related disorders (Brewin et al. 2010) but less explored in BDD.

### 1.2 Mental Imagery

Mental imagery refers to cognitive representations of perceptual experience (Kosslyn et al. 2006; Pearson et al. 2015), most commonly associated with contexts such as dreams, memories or daydreaming, but also within broad mental activities such as goal‐setting and emotion regulation (Kosslyn and Jolicoeur 2021). Unlike direct perception from sensory organs, mental imagery (1) involves subjective semblance of sensory input, which can involve the full range of sensory modalities, with visual being the most common (Pearson et al. 2015); (2) may incorporate perceived events or representations not previously seen by the senses; (3) represents concrete or abstract scenarios, which can be static or dynamic (in movement or temporal sequence); (4) may be deliberately constructed or involuntarily generated. From a neuroscientific perspective, visual imagery and visual perception share overlapping neurocircuitry, which may partly explain the ‘as‐if’ reality quality associated with imagery (Ji et al. 2019; Pearson et al. 2015).

Self‐relevant forms of imagery refer to multi‐sensory images in which the self (or parts of the self) is represented as the object or agent within a mental scene (Blackwell 2020; Brewin et al. 2010). These types of imagery often carry high emotional salience, are perceived as involuntary and intrusive and are therefore most relevant to emotional psychopathology. Involuntary imagery has been described under numerous terms, including unwanted mental intrusions (Pascual‐Vera et al. 2019), intrusive mental images (Brewin et al. 2010), intrusive memories (Iyadurai et al. 2019), spontaneous imagery (Osman et al. 2004) and ‘flashforwards’ (Lau‐Zhu, Stacey, et al. 2024). We do not focus on neutral, non‐autobiographical imagery—often assessed with cognitive tests—because these do not appear to be reliably linked with emotional symptoms (Di Simplicio et al. 2019, 2016).

Imagery is proposed to causally influence physical or emotional symptoms such as distress and dissociative reactions. Due to the recurrent intrusive presentation of emotionally upsetting content (Pearson et al. 2015), imagery has been proposed to be a key maintenance factor in many psychological disorders (Brewin et al. 2010; Chapman et al. 2020), especially where the content reflects early aversive memories or negative core beliefs. Intrusive images can prompt attempts at cognitive avoidance or suppression, which may reciprocally maintain intrusions and associated negative thinking (Ji et al. 2019).

Imagery‐based psychological interventions involve generating and/or manipulating mental imagery to address mental health issues. These techniques may be applied as standalone interventions, such as imagery rescripting (ImRs), guided imagery and imaginal exposure (Saulsman et al. 2019), or as components of evidence‐based interventions such as CBT, exposure with response prevention (ERP), metacognitive therapy (MCT) and eye movement desensitisation and reprocessing (EMDR). Imagery‐based interventions are integral to several manualised versions of CBT for post‐traumatic stress disorder (PTSD), anxiety disorders, depression and phobias (Kip et al. 2023), often involving the replaying or reprocessing of aversive memories or exposure to threatening stimuli without the requirement for their physical presence (Stopa 2009). In BDD, imagery‐based approaches often involve appearance‐related memories or stimuli associated with the individual's perceived flaws (Veale et al. 2025).

### 1.3 Mental Imagery in BDD

A cognitive‐behavioural model of BDD, conceptualised by Veale (2004), (Veale et al. 1996), has seen successful application to therapeutic efforts in BDD and other body image‐related problems in adults (Harrison et al. 2016). It explains how BDD develops within a constellation of maintenance factors related to body dissatisfaction. Several other theoretical frameworks outside cognitive‐behavioural approaches address BDD and body dissatisfaction, including sociocultural (Mills et al. 2022) and biopsychosocial models (Möllmann et al. 2024; Neziroglu and Barile 2017). Nevertheless, these broadly converge on core themes of self‐focused attention, preoccupation with perceived flaws and attempts to modify or monitor the body as key psychological processes. The model by Veale (2004), Veale et al. (1996) is especially relevant in this review, as it explicitly incorporates imagery as a key driver and is supported by emerging empirical research and clinical application to BDD and body dissatisfaction.

**FIGURE 1:**
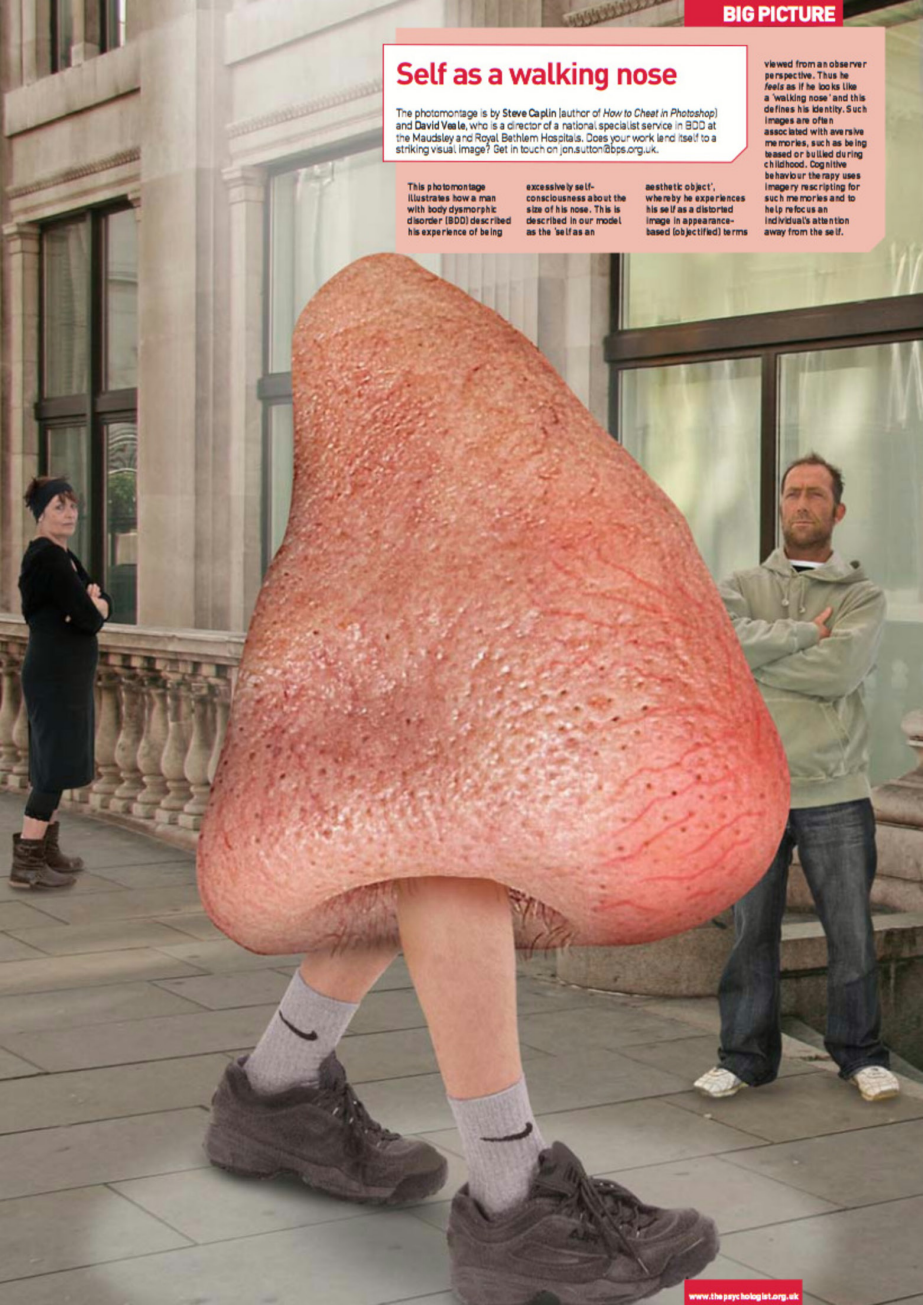
Pictorial illustration of a ‘self as a walking nose’ example of the felt impression in BDD, reproduced with author's permission from https://www.bps.org.uk/psychologist/big‐picture‐self‐walking‐nose.

**FIGURE 2:**
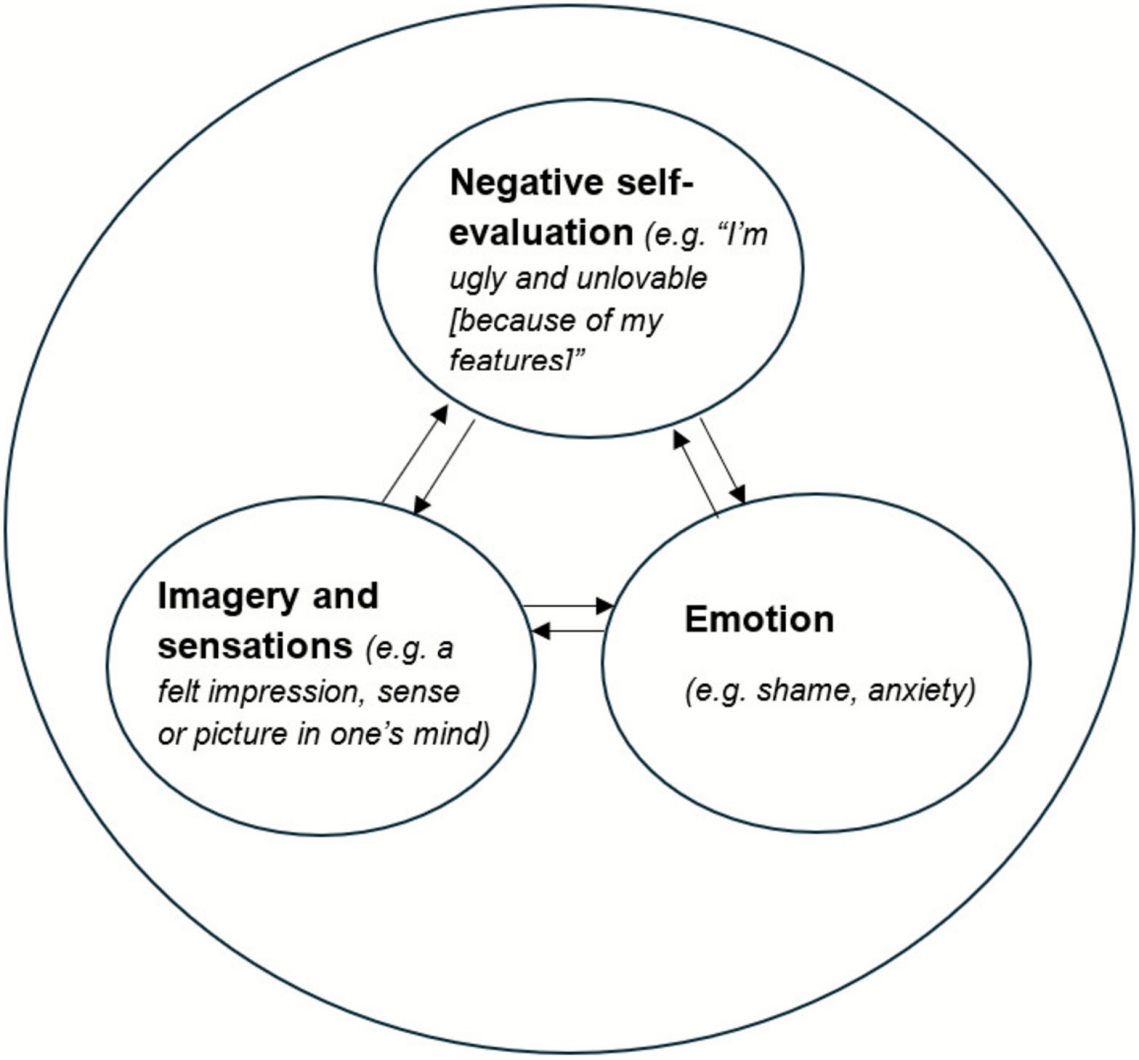
Diagrammatic representation of the felt impression within the ‘self as an aesthetic object’ model of BDD.

Core to Veale's model is the notion of ‘self as an (ugly) aesthetic object’, that is, extreme self‐focused attention and self‐consciousness due to exaggerated perceived value in judging the self by the perceived defect (Veale et al. 2025). This is presented pictorially in Figure 1, illustrating an example preoccupation of ‘self as a walking nose’. In brief, individuals experience an intrusive mental image or ‘felt sense’ that portrays this perceived (potential) ugliness or defectiveness, often with a deep sense of shame. The imagery might consist of one or more sensory domains including visual, sensory or olfactory (Osman et al. 2004). Individuals' attention may become fixated on this ‘felt sense’, from which their appearance evaluations base heavily. This fixation prompts self‐focused attention towards the perceived defect, negative appraisals of one's body image, negative emotions (especially of shame and disgust) and comparisons between the perceived and ideal (i.e., an aesthetic) self. Instances of verifying external representations of appearance (e.g., through mirror checking, photos or thinking about one's image), comparing and ruminating, serve to further maintain the negative self‐image, reinforcing the processing of the self as an aesthetic object. The cognitive‐behavioural model is presented graphically in Figure 2.

Imagery is relevant to two cognitive processes of BDD within Veale's model: (1) appearance‐related intrusive thoughts, (2) appearance‐related preoccupations. Both intrusions and preoccupations can take the form of verbal thinking (i.e., words and sentences), mental imagery or a combination. Intrusions are brief, involuntary experiences (e.g., ‘my nose is huge’), whereas preoccupations involve more sustained ruminative or comparative thinking (e.g., attending to images of their nose in different settings). In Veale's model, the combination of these processes reinforces the distress and safety‐seeking behaviours displayed in BDD. Some cognitive accounts propose that imagery‐based processing, relative to purely verbal thinking, amplifies emotions and motivations to act (Holmes et al. 2008; Ji et al. 2019).

Over time, greater frequency and sophistication of behaviours are developed, aimed at altering, hiding or improving one's appearance, such as camouflaging, socially withdrawing or appearance‐altering procedures. As such, behaviours that hallmark BDD are understood to serve a safety‐seeking function, through monitoring the source of their distress, gaining reassurance about their appearance or attempting to remove/hide the perceived flaw or themselves from situations others would notice these flaws (Veale et al. 1996).

Empirical exploration of imagery in BDD has risen following an initial study by Osman et al. (2004). In a sample of 18 BDD patients, 94% reported imagery that was more spontaneous, intrusive, recurrent, frequent and vivid than a control group. These images commonly comprised specific body image flaws and were associated with stressful memories related to appearance (e.g., involving bullying, teasing and/or perceived rejection). Since this study, the role of imagery in BDD has been increasingly explored, particularly in relation to disorder maintenance/exacerbation (e.g., Brennan et al. 2023) and therapeutic interventions (e.g., Ghaderi et al. 2022; Veale, Anson, et al. 2014).

Although systematic reviews of mental imagery have been conducted in other psychopathology‐related areas, including worry (Stavropoulos et al. 2024), psychological trauma (Iyadurai et al. 2019), suicide and self‐harm (Lawrence et al. 2023), social anxiety (Chapman et al. 2020), bipolar disorder (Petit et al. 2021) and children and adolescents (Schwarz et al. 2020), no such review exists for BDD. Given the proposed importance of imagery in theoretical models and psychological interventions for BDD, there is a strong and timely rationale for a comprehensive synthesis of empirical work exploring its phenomenology, underpinning mechanisms and treatment implications in BDD.

### 1.4 Aim of the Review

We aimed to synthesise the literature of mental imagery in relation to the understanding and treatment of BDD and identify theoretical and clinical implications and areas for future research. Our focus is on self‐relevant, emotionally charged mental imagery and their contributing mechanisms to BDD symptomology, as these appear particularly relevant to emotional psychopathology more broadly relative to non‐emotional neutral imagery (Di Simplicio et al. 2016, 2019). Our central questions were: *what are the characteristics of mental imagery (relevant to the self) in individuals with BDD?* and *what are the hypothesised mechanisms by which such imagery contributes to the development, persistence and amelioration of BDD?*

## 2 Method

### 2.1 Protocol and Reporting Guidelines

The review protocol was preregistered at PROSPERO on 13 September 2023 (ID CRD42023450339). Minor deviations to the original protocol were implemented, detailed in Appendix A. The Preferred Reporting Items for Systematic Reviews and Meta‐Analysis (PRISMA) reporting guidelines for systematic reviews (Page et al. 2021) were followed.

### 2.2 Eligibility Criteria

Inclusion criteria for eligibility were: (1) studies focussing on BDD, body dissatisfaction or body‐image concern as the primary psychological construct; (2) studies involving mental imagery as a component of the research; (3) primary studies comprising quantitative, qualitative or mixed‐method research data. We included imagery‐based intervention studies as these have the potential to inform the mechanisms through which imagery maintains BDD symptoms and which are presumably targeted by such interventions. We also included individuals with body dissatisfaction (but without necessarily a diagnosis of BDD), in line with a mechanistic perspective which considers psychopathology as a continuum from subclinical vulnerability states to clinical disorders (Wittchen et al. 2014), including for body‐image psychopathology. This wider scope also allowed us to be more inclusive of the literature in an emerging field in a first review of this topic.

Exclusion criteria were: (1) literature primarily concerned with feeding and eating disorders, due to their distinct psychopathology compared to BDD; (2) publications unsuitable for primary data extraction, such as reporting data secondarily (e.g., reviews of all types, commentaries and editorials), non‐standard sources (e.g., websites) and those not published in a peer‐reviewed journal (e.g., books and pre‐prints).

### 2.3 Information Sources

The following databases were searched on 14 February 2025: MEDLINE, EMBASE, PsycINFO, CINAHL, Scopus and Web of Science Core Collection. No restrictions and limitations (e.g., language, publication date and population) were applied.

### 2.4 Search Strategy

Keywords were adopted to capture the concepts of mental imagery and BDD/body dissatisfaction. The same concepts were applied to all databases shown in Appendix B with slight variations due to different search engines.

### 2.5 Selection and Data Extraction

Duplicate records were cross‐checked and removed. Publication titles and abstracts (*N* = 3547) were screened against eligibility criteria. Then, 20% (*n* = 709) were stratified randomly by year of publication and screened by two independent researchers (SH and MW), reaching 99.4% agreement (Cohen's Kappa = 0.94, 95% CI: 88–99), denoting ‘almost perfect agreement’ (McHugh 2012). All remaining records were screened by the lead author.

Full‐text publications (*N* = 124) were screened further, 25% (*n* = 31) by two researchers (SH and MW) independently, from which seven disagreements were detected and resolved with the supervising co‐authors (ALZ and MH). With agreements resolved, the remaining were assessed by the lead author. Data were extracted by two researchers (SH and MW) for 50% (*n =* 17) of records, which were compared to check for agreement and discrepancies, of which none were encountered. The remaining records (*n* = 17) were extracted by the lead author.

### 2.6 Quality Assessment

Studies were appraised using the Mixed‐Method Appraisal Tool (MMAT; Hong et al. 2018), a reliable indicator of validity, reliability and generalisability in qualitative, quantitative and mixed‐methods studies (Pace et al. 2012). We expected a combination of qualitative and quantitative approaches, given the importance of subjective experience in characterising and measuring mental imagery, especially in populations (Di Simplicio et al. 2019, 2016). As the MMAT recommends against overall quality ratings, tabulated ratings for each MMAT criterion are presented, with an accompanying description of trends (Section 3.1.1) as recommended by Hong et al. (2018).

### 2.7 Synthesis Method

Given the mixed‐method evidence base, a Meta‐Integration was performed using the convergent segregated approach outlined by the Joanna Briggs Institute (2014). The process involved (1) synthesis of quantitative data; (2) synthesis of qualitative data and (3) integration of the syntheses, reporting instances where results complement and conflict. For mixed‐methods studies, the quantitative and qualitative findings were analysed separately. As it is common with qualitative approaches (Levitt et al. 2022), Meta‐Integration acknowledges the role of researcher subjectivity; thus, author position statements are detailed in Appendix C to contextualise the researchers' interpretation.

For quantitative studies, a Narrative Synthesis was performed as outlined by Popay et al. (2006). This process involved (1) organising extracted data into meaningful categories; (2) grouping findings by themes or patterns and (3) exploring and reporting relationships in the data. A meta‐analytic approach was precluded due to study heterogeneity in terms of designs and measures (see Section 3.1.3).

For qualitative studies, Thematic Synthesis was performed as described by Thomas and Harden (2008). This process involved three steps: (1) coding text from eligible studies; (2) developing descriptive themes and (3) generating analytical themes. All contents of the results sections within reviewed studies were classified as data. For mixed methods studies, this occurred after separation from quantitative results.

The quantitative and qualitative syntheses (Sections 3.2 and 3.3) will be structured to describe patterns of data, whereas the meta‐integration (Section 3.1.3.) will be structured by review questions (including imagery characteristics and hypothesised mechanisms) to connect the findings across the evidence base back to the review questions. Following completion of quality appraisal (Section 3.1.1), we identified that quantitative studies were generally of medium quality and qualitative studies generally of high quality (though still weak for deriving causal claims). As weighting findings by quality was deemed unlikely to produce meaningful distinctions across studies, we opted to interpret the overall emerging picture. This approach aligns with both Narrative Synthesis (Popay et al. 2006) and Thematic Synthesis (Thomas and Harden 2008).

## 3 Results

### 3.1 Study Selection

**FIGURE 3:**
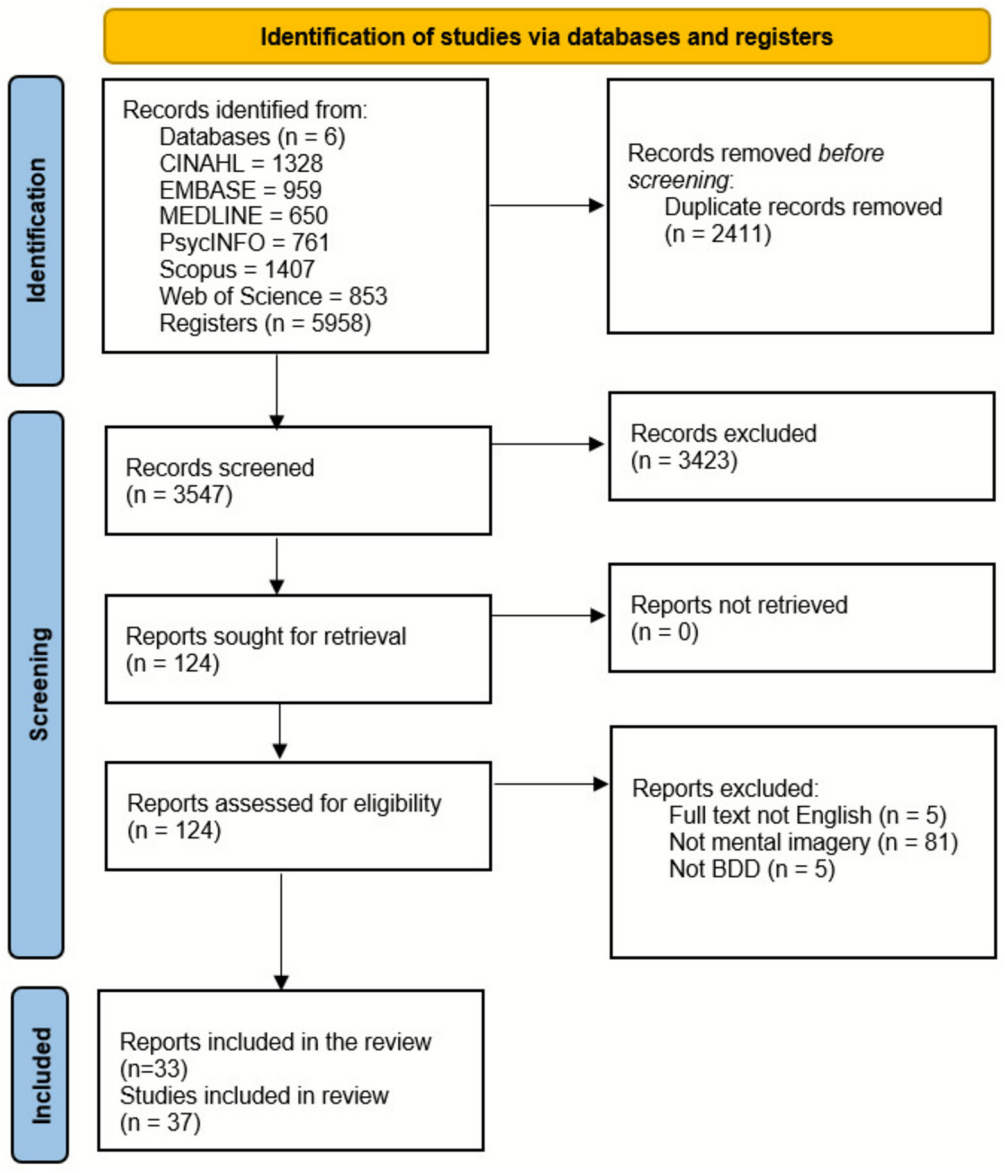
PRISMA flow diagram.

From 5958 records identified from the initial searches, 3547 abstracts underwent screening, with 124 publications selected for full‐text assessment. Thirty‐seven studies were included from 33 records (as some records included more than one study); 29 quantitative, six qualitative and two mixed in design. Study selection is presented graphically in Figure 3.

The included records spanned the publication years from 1997 to 2024, most commonly 2022 (*n* = 6), followed by 2014, 2015, 2023 and 2024 (each *n* = 3). Research was contributed from several countries, although biased towards the ‘Global North’, most commonly the United Kingdom (*n* = 14), Spain (*n* = 5), the United States (*n* = 3) and Australia (*n* = 3). Germany, Iran and China each contributed two studies. Japan, Sweden and Singapore each contributed one study. Additionally, one UK‐based study included participants from India, the United Kingdom and United States (Craythorne et al. 2022) and one Spain‐based study included participants from Argentina, Iran, Israel, Italy, Portugal, Spain and Turkey (Pascual‐Vera et al. 2022).

Seventeen papers involved specific body areas of concern, the most frequent being the skin (*n* = 8), face, hair, nose (each *n* = 6), eyes, genitalia and legs (each *n* = 5). All non‐intervention studies (*n* = 25) were cross‐sectional. Of the intervention studies (*n* = 12), 3 were randomised controlled trials (RCT) with the remainder (*n* = 9) non‐randomised. Interventions comprised ImRs as stand‐alone (*n* = 3; one RCT, two case series), CBT incorporating ImRs (*n* = 2; one RCT and one single case experimental design or SCED), MCT (*n* = 1, case series), EMDR (*n* = 3; one SCED and two case series), mindfulness‐based cognitive therapy (MBCT, *n* = 1; one RCT) and ERP (*n* = 1 case series).

#### 3.1.1 Quality Appraisal

Quantitative studies varied in quality. Most failed to adequately demonstrate using a representative sample for the BDD population, adequate control of appropriate confounding variables or justification of the psychometrics used. These weaknesses were compounded by the large number of BDD measurement tools used (Section 3.1.3), hampering comparability between studies. The three randomised‐controlled trials (RCTs, Ghaderi et al. 2022; Gu and Zhu 2023; Veale, Anson, et al. 2014) were of high quality, although intervention blinding consistently scored poorly, a common issue in psychological interventions. Other than the RCTs, intervention studies were generally poorer in quality than experimental studies, regularly displaying poor explanation of their procedure, sampling rationale and process of accounting for confounds.

Qualitative studies were generally of higher quality. No studies received a ‘fail’ on any criterion, although some suffered from underexplained methods sections, leading to obscurity in data collection methods (Brennan et al. 2023; Silver and Reavey 2010; Stechler and Henton 2022) and coherence between data and their interpretations (Cooper and Osman 2007; Craythorne et al. 2022). Of the two mixed method studies, one scored ‘fairly’ with a lack of clarity in the qualitative methodology (Osman et al. 2004); the other scored ‘poorly’ due to significant underreporting of methods and findings (Ghaderi et al. 2022). Full quality assessment outcomes are displayed in Table 1.

**TABLE 1:** Quality assessment using mixed methods appraisal tool (MMAT).

	Brennan et al. (2023)	Cooper and Osman (2007)	Craythorne et al. (2022)	Onden‐Lim and Grisham (2013)	Silver and Reavey (2010)	Stechler and Henton (2022)	Ghaderi et al. (2022)—Study 3	Gu and Zhu (2023)	Veale, Anson, et al. (2014)	Brown et al. (1997)	Chuah and Suendermann (2024)	Dziegielewski and Wolfe (2000)
Screening questions												
S1. Are there clear research questions?	**✔**	**✔**	**✔**	**✔**	**✔**	**✔**	**✔**	**✔**	**✔**	✘	**✔**	**✔**
S2. Do the collected data allow to address the research questions?	**✔**	**✔**	**✔**	**✔**	**✔**	**✔**	**✔**	**✔**	**✔**	✘	**✔**	**✔**
Qualitative studies												
1.1. Is the qualitative approach appropriate to answer the research question?	**✔**	**✔**	**✔**	**✔**	**✔**	**✔**						
1.2. Are the qualitative data collection methods adequate to address the research question?	—	**✔**	**✔**	**✔**	—	—						
1.3. Are the findings adequately derived from the data?	**✔**	**✔**	**✔**	—	**✔**	**✔**						
1.4. Is the interpretation of results sufficiently substantiated by data?	**✔**	**✔**	**✔**	—	**✔**	**✔**						
1.5. Is there coherence between qualitative data sources, collection, analysis and interpretation?	**✔**	—	—	**✔**	**✔**	**✔**						
Quantitative randomised studies												
2.1. Is randomisation appropriately performed?							**✔**	**✔**	**✔**			
2.2. Are the groups comparable at baseline?							**✔**	**✔**	**✔**			
2.3. Are there complete outcome data?							**✔**	**✔**	**✔**			
2.4. Are outcome assessors blinded to the intervention provided?							✘	—	✘			
2.5 Did the participants adhere to the assigned intervention?							**✔**	**✔**	**✔**			
Quantitative non‐randomised studies												
3.1. Are the participants representative of the target population?										✘	✘	✘
3.2. Are measurements appropriate regarding both the outcome and intervention (or exposure)?										✘	**✔**	**✔**
3.3. Are there complete outcome data?										✘	**✔**	✘
3.4. Are the confounders accounted for in the design and analysis?										✘	—	✘
3.5. During the study period, is the intervention administered (or exposure occurred) as intended?										**✔**	**✔**	**✔**
Quantitative descriptive studies												
4.1. Is the sampling strategy relevant to address the research question?												
4.2. Is the sample representative of the target population?												
4.3. Are the measurements appropriate?												
4.4. Is the risk of nonresponse bias low?												
4.5. Is the statistical analysis appropriate to answer the research question?												
Mixed method studies												
5.1. Is there an adequate rationale for using a mixed methods design to address the research question?												
5.2. Are the different components of the study effectively integrated to answer the research question?												
5.3. Are the outputs of the integration of qualitative and quantitative components adequately interpreted?												
5.4. Are divergences and inconsistencies between quantitative and qualitative results adequately addressed?												
5.5. Do the different components of the study adhere to the quality criteria of each tradition of the methods involved?												

	Farrar et al. (2015)	Ghaderi et al. (2022)—Study 2	Grocholewski et al. (2018)	Hamamoto et al. (2024)	McKay et al. (1997)	Moreno‐Domínguez et al. (2012)	Pascual‐Vera et al. (2022)	Pouladi et al. (2024)	Rabiei et al. (2012)	Ritter and Stangier (2016)	Sündermann et al. (2016)	Van den Berg and Thompson (2007)
Screening questions												
S1. Are there clear research questions?	**✔**	**✔**	**✔**	**✔**	**✔**	**✔**	**✔**	**✔**	**✔**	**✔**	**✔**	**✔**
S2. Do the collected data allow to address the research questions?	—	**✔**	**✔**	**✔**	✘	**✔**	**✔**	**✔**	—	**✔**	**✔**	**✔**
Qualitative studies												
1.1. Is the qualitative approach appropriateto answer the research question?												
1.2. Are the qualitative data collection methods adequate to address the research question?												
1.3. Are the findings adequately derived from the data?												
1.4. Is the interpretation of results sufficiently substantiated by data?												
1.5. Is there coherence between qualitative data sources, collection, analysis and interpretation?												
Quantitative randomised studies												
2.1. Is randomisation appropriately performed?												
2.2. Are the groups comparable at baseline?												
2.3. Are there complete outcome data?												
2.4. Are outcome assessors blinded to the intervention provided?												
2.5 Did the participants adhere to the assigned intervention?												
Quantitative non‐randomised studies												
3.1. Are the participants representative of the target population?	✘	✘	**✔**	✘	✘	✘	**✔**	✘	✘	✘	**✔**	**✔**
3.2. Are measurements appropriate regarding both the outcome and intervention (or exposure)?	—	**✔**	**✔**	**✔**	**✔**	**✔**	**✔**	✘	✘	**✔**	✘	✘
3.3. Are there complete outcome data?	**✔**	**✔**	**✔**	**✔**	**✔**	**✔**	**✔**	**✔**	✘	**✔**	**✔**	**✔**
3.4. Are the confounders accounted for in the design and analysis?	✘	**✔**	**✔**	—	✘	—	**✔**	**✔**	✘	—	**✔**	—
3.5. During the study period, is the intervention administered (or exposure occurred) as intended?	**✔**	**✔**	**✔**	**✔**	**✔**	—	**✔**	**✔**	**✔**	**✔**	**✔**	**✔**
Quantitative descriptive studies												
4.1. Is the sampling strategy relevant to address the research question?												
4.2. Is the sample representative of the target population?												
4.3. Are the measurements appropriate?												
4.4. Is the risk of nonresponse bias low?												
4.5. Is the statistical analysis appropriate to answer the research question?												
Mixed method studies												
5.1. Is there an adequate rationale for using a mixed methods design to address the research question?												
5.2. Are the different components of the study effectively integrated to answer the research question?												
5.3. Are the outputs of the integration of qualitative and quantitative components adequately interpreted?												
5.4. Are divergences and inconsistencies between quantitative and qualitative results adequately addressed?												
5.5. Do the different components of the study adhere to the quality criteria of each tradition of the methods involved?												

	Veale and Riley (2001)	Veale et al. (2016)	Willson et al. (2016)	Giraldo‐O'Meara and Belloch (2018)	Giraldo‐O'Meara and Belloch (2019)—Study 1	Giraldo‐O'Meara and Belloch (2019)—Study 2	Giraldo‐O'Meara and Belloch (2019)—Study 3	Liu et al. (2023)	Onden‐Lim and Grisham (2012)	Pascual‐Vera et al. (2019)	Veale et al. (2015)	Ghaderi et al. (2022)—Study 1	Osman et al. (2004)
Screening questions													
S1. Are there clear research questions?	—	**✔**	**✔**	**✔**	**✔**	**✔**	**✔**	**✔**	**✔**	**✔**	**✔**	**✔**	**✔**
S2. Do the collected data allow to address the research questions?	**✔**	**✔**	**✔**	**✔**	**✔**	**✔**	**✔**	**✔**	**✔**	**✔**	**✔**	**✔**	**✔**
Qualitative studies													
1.1. Is the qualitative approach appropriate to answer the research question?													
1.2. Are the qualitative data collection methods adequate to address the research question?													
1.3. Are the findings adequately derived from the data?													
1.4. Is the interpretation of results sufficiently substantiated by data?													
1.5. Is there coherence between qualitative data sources, collection, analysis and interpretation?													
Quantitative randomised studies													
2.1. Is randomisation appropriately performed?													
2.2. Are the groups comparable at baseline?													
2.3. Are there complete outcome data?													
2.4. Are outcome assessors blinded to the intervention provided?													
2.5 Did the participants adhere to the assigned intervention?													
Quantitative non‐randomised studies													
3.1. Are the participants representative of the target population?	✘	—	—										
3.2. Are measurements appropriate regarding both the outcome and intervention (or exposure)?	✘	**✔**	**✔**										
3.3. Are there complete outcome data?	**✔**	**✔**	**✔**										
3.4. Are the confounders accounted for in the design and analysis?	✘	**✔**	—										
3.5. During the study period, is the intervention administered (or exposure occurred) as intended?	**✔**	**✔**	**✔**										
Quantitative descriptive studies													
4.1. Is the sampling strategy relevant to address the research question?				**✔**	**✔**	**✔**	**✔**	—	—	**✔**	**✔**		
4.2. Is the sample representative of the target population?				**✔**	**✔**	**✔**	**✔**	**✔**	✘	—	✘		
4.3. Are the measurements appropriate?				**✔**	**✔**	**✔**	**✔**	**✔**	**✔**	**✔**	**✔**		
4.4. Is the risk of nonresponse bias low?				**✔**	**✔**	**✔**	**✔**	—	**✔**	**✔**	**✔**		
4.5. Is the statistical analysis appropriate to answer the research question?				**✔**	**✔**	**✔**	**✔**	**✔**	**✔**	**✔**	**✔**		
Mixed method studies													
5.1. Is there an adequate rationale for using a mixed methods design to address the research question?												**✔**	**✔**
5.2. Are the different components of the study effectively integrated to answer the research question?												✘	**✔**
5.3. Are the outputs of the integration of qualitative and quantitative components adequately interpreted?												✘	**✔**
5.4. Are divergences and inconsistencies between quantitative and qualitative results adequately addressed?												✘	—
5.5. Do the different components of the study adhere to the quality criteria of each tradition of the methods involved?												—	—

*Note:* Boxes are marked ‘✔’ to indicate ‘yes’, ‘‐’to indicate ‘unsure/partial’ or ‘✘’ to indicate ‘no’ to MMAT criteria. Boxes in grey are where the item was not applicable due to a different study design—the MMAT requires only items relevant to the study's design be scored.

#### 3.1.2 Demographic Data

All studies focused on the working‐age adults (18–65 years): only one included a participant below age 18—a 16 year‐old (Rabiei et al. 2012). Participants were typically aged 18–60, with mean age varying significantly across studies, with the lowest mean at 19 years (Onden‐Lim and Grisham 2013) and the oldest at 42 years (Veale et al. 2015). The oldest participants across studies were 64 years old (Pascual‐Vera et al. 2022).

Around half of studies (*n* = 18; 48.6%) did not report ethnicity data. When reported, most studies examined a predominant ethnic group, which was either White British/other in the ‘Global North’ (Veale et al. 2015) or Chinese or Singaporean Asian (Chuah and Suendermann 2024; Liu et al. 2023). Three studies (Van den Berg and Thompson 2007; Pascual‐Vera et al. 2022; Onden‐Lim and Grisham 2013) included varied ethnicities with no predominant group. Some studies also reported marital status (*n* = 10), education level (*n* = 9), socioeconomic status (*n* = 5), employment status (*n* = 5), body mass index or BMI (*n* = 5) and mental health comorbidity (*n* = 6).

#### 3.1.3 Measurements

##### 3.1.3.1 BDD

Twenty instruments measured BDD symptomology across studies, including those broadly designed for BDD, body dissatisfaction and eating disorders. Most common were the BDD‐YBOCS (Phillips et al. 1997, *n* = 11) and Body Dysmorphic Disorder Diagnostic Module (BDDDM; Phillips 2017, *n* = 4). Nineteen included participants with a formal diagnosis of BDD, established using the Structured Clinical Interview for DSM‐IV and DSM‐IV‐TR (SCID‐IV; SCID‐IV‐TR; Glasofer et al. 2015; *n* = 7); BDD‐YBOCS (*n* = 5); checking against DSM‐IV diagnostic criteria (*n* = 4); Body Dysmorphic Disorder Questionnaire (BDDQ; Brohede et al. 2013, *n* = 2) or BDDDM (*n* = 2).

##### 3.1.3.2 Imagery

Seven self‐report measures were used to assess mental imagery: Thought Fusion Inventory (TFI; Wells and Carter 2001), Questionnaire of Unpleasant Intrusive Thoughts (QUIT; Pascual‐Vera et al. 2019), Intrusive Visual Imagery Scale (IVIS), Appearance Intrusions Questionnaire (AIQ; Giraldo‐O'Meara and Belloch 2019), Mirror‐gazing: Cognition and Affect Rating Scale (MG‐CARS; Windheim et al. 2011) and EMDR treatment scales: Subjective Units of Disturbance (SUD) and Validity of Cognition (VOC) (Shapiro 2001). These measures captured different aspects of imagery. The AIQ (Giraldo‐O'Meara and Belloch 2019) specifically measured frequency of unwanted appearance‐related intrusive thoughts, images, impulses and sensations. Two instruments (IVIS, QUIT) measured imagery content, modality, vividness and affective change. Two instruments (MG‐CARS, EMDR SUD and VOC) measured only affective change. The TFI measured metacognitive beliefs about the meaning, significance and dangerousness of intrusive thoughts (Rabiei et al. 2012).

Nine studies used semi‐structured interviews to explore imagery. Three based their interviews on the same schedule pioneered by Ann Hackmann (Osman et al. 2004; Onden‐Lim and Grisham 2012; Grocholewski et al. 2018). To help evoke imagery during interviews, one study invited participants to bring photos of themselves from childhood, using these to recollect memories and present‐day appearance comparisons (Silver and Reavey 2010). Another asked participants to complete an autobiographical writing task about an appearance‐related memory (Craythorne et al. 2022).

Seven experimental studies and nine intervention studies did not measure imagery directly, but incorporated imagery as a component of the experimental or therapeutic paradigms and primarily measured another construct within BDD. The only intervention studies including imagery as an outcome examined EMDR (Ghaderi et al. 2022), MCBT study (Gu and Zhu 2023) or MCT (Rabiei et al. 2012).

### 3.2 Quantitative Synthesis

Thirty‐one studies were included in the quantitative synthesis. These were organised into: non‐intervention (*n* = 19), which included experimental manipulations of imagery and/or techniques; intervention studies (*n* = 12), which included those manipulating imagery in clinical settings. Included studies are summarised in Tables 2 and 3 respectively.

**TABLE 2:** Summary of non‐intervention studies included in quantitative synthesis.

Authors and years of publication	Population (*N*, age, sex and formal diagnosis^a^ Y/N)	Sample description	Comparison groups, treatment and/or clinical group (if applicable)	BDD measure(s)	Imagery measure(s); aspects of imagery measured	Main findings^b^
Chuah and Suendermann (2024)	*N* = 63 Mean age: 21.22 Sex: *M* = 28, *F* = 35 Diagnosis: No	Non‐clinical undergraduate population; body dissatisfaction not specifically screened, no formal BDD diagnosis.	Mirror gazing with high self‐focus vs. Mirror gazing with low self‐focus	BIDQ (Cash et al. 2004), VAS for body dissatisfaction	VAS; vividness and emotional quality of appearance‐related image	High self‐focused attention did not increase the vividness or emotional negativity of appearance‐related imagery
Farrar et al. (2015)	*N* = 66 Mean age: Positive imagery = 20.18, Negative imagery = 19.73 Sex: *M* = 0, *F* = 66 Diagnosis: No	University female students with elevated body dissatisfaction (BSQ ≥ 95); non‐clinical but high body‐image concern	Positive imagery manipulation vs. negative imagery manipulation	BSQ (Cooper et al. 1987)	Idiographic 100‐point scale; imagery vividness and positive/negative affect related to image	Negative self‐images reduced self‐esteem, body satisfaction and clarity of self‐concept, while increasing negative affect. Positive self‐images improved explicit self‐esteem and body satisfaction but did not affect clarity of self‐concept.
Ghaderi et al. (2022)—Study 1^c^	*N* = 31 Mean age: 27.8 Sex: *M* = 0, *F* = 31 Diagnosis: No	Female university students with varying levels of body dissatisfaction; non‐clinical sample	N/A	BSQ and IBSS	VAS; vividness and relative negative affect of mental imagery.	All participants experienced body‐related mental images, either positive or negative. Body dissatisfaction was linked to the frequency and negative nature of images, but not to positive images or general mental imagery.
Giraldo‐O'Meara and Belloch (2019)—Study 1	*N* = 410 Mean age: 23.5 Sex: *M* = 103, *F* = 307 Diagnosis: No	Non‐clinical sample of Spanish undergraduate students, predominantly female, general population	No comparison group.	None.	AIQ; intrusiveness of appearance‐related imagery	Confirmed a five‐factor structure for appearance intrusions in support of the AIQ.
Giraldo‐O'Meara and Belloch (2019)—Study 2	*N* = 583 Mean age: 29.8 Sex: *M* = 166, *F* = 417 Diagnosis: No	Non‐clinical sample, diverse education and socioeconomic backgrounds	No comparison group	None.	AIQ; intrusiveness of appearance‐related imagery	Over 90% of respondents reported experiencing at least one appearance‐related intrusive thought, with women reporting significantly higher total scores than men, except in the ‘urge to do something’ subscale.
Giraldo‐O'Meara and Belloch (2019) ‐ Study 3	*N* = 583 Mean age: BDD risk = 22.59, No BDD risk = 30.91 Sex: *M* = 147, *F* = 359 Diagnosis: Yes	Non‐clinical sample, diverse education and socioeconomic backgrounds	At risk of BDD vs. not at risk of BDD	BDDQ; MDBSRQ—Appearance scales (Cash 2000)	AIQ; intrusiveness of appearance‐related imagery	Strong correlations were found between AIQ, BDD‐related symptoms and body image concerns, with the highest associations seen with the body areas satisfaction subscale of the MBSRQ. Individuals at risk for BDD reported significantly more appearance‐related intrusive thoughts than those at no risk.
Giraldo‐O'Meara and Belloch (2018)	*N* = 40 Mean age: BDD = 23.10, High risk for BDD = 22.87, Low risk for BDD = 24.00 Sex: *M* = 21, *F* = 19 Diagnosis: Yes	BDD‐screened subgroup (*n* = 68 met BDDQ criteria); general population with medium socioeconomic status; non‐clinical participants included if they reported a highly upsetting appearance‐related thought in the past 3 months	BDD vs. High‐risk for BDD vs. low‐risk for BDD	BDD‐YBOCS, BDDQ and SCID‐IV	AIQ; intrusiveness of appearance‐related imagery	Most participants experienced unwanted intrusive thoughts about their appearance. Individuals used both positive (cognitive restructuring, distraction) and negative (comparing themselves to others) strategies to control these intrusions.
Grocholewski et al. (2018)	N = 63 Mean age: BDD = 35.26, BIID = 50.73, Control = 38.95 Sex: *M* = 35, *F* = 28 Diagnosis: Yes	BDD participants recruited from university outpatient and psychosomatic inpatient units; BIID participants recruited from online forums and specialised units; controls recruited from community	BDD vs. Body Integrity Identity Disorder (BIID) vs. non‐clinical controls	BDDDM and BIID screening questionnaire	Semi‐structured interview adapted from Osman et al. (2004); BDD‐imagery characteristics	Individuals with BDD and BIID both experience frequent mental images. BDD participants experienced them as more intrusive and distressing compared to BIID participants, who were more likely to find them sexually arousing.
Hamamoto et al. (2024)	*N* = 28 Mean age: Mirror exposure = 22.1, Mental imagery = 22.8 Sex: *M* = 0, *F* = 28 Diagnosis: No	Non‐clinical sample recruited from university and local community; no psychiatric disorders or current weight‐loss plans	Mirror exposure vs. mental imagery	EDI‐2 (Tachikawa et al. 2004), Body image silhouettes	None used as outcome measures. ‘Self‐generated’ mental imagery of the self was a compared intervention to mirror exposure	Mental imagery, rather than mirror exposure, was found to be more effective in reducing perceptual disturbances and enhancing visual processing.
Liu et al. (2023)	*N* = 5909 Mean age: 19.87 Sex: *M* = 2730, *F* = 3179 Age range: 18–32 Diagnosis: No	Non‐clinical sample of Chinese college students	N/A: This large survey involved no interventions or treatment groups.	SDBPS (Berscheid et al. 1973)	IVIS; presence and intensity of intrusive imagery experience	Positive correlation between body dissatisfaction and smartphone addiction, with intrusive imagery and fear of negative evaluation acting as mediators in this relationship.
Moreno‐Domínguez et al. (2012)	*N* = 31 Mean age: 20.12 Sex: *M* = 0, *F* = 31 Diagnosis: No	Women screened for body dissatisfaction, not on weight‐loss programmes	Pure (unguided) mirror exposure vs. guided mirror exposure vs. mental imagery exposure	BSQ and EAT (Mintz and O'Halloran 2000), VAS for beauty versus ugliness	Idiographic thoughts checklist; for negative cognitions related to body‐related imagery	Pure mirror exposure was the most effective intervention for reducing subjective discomfort and sustaining reductions in negative thoughts and body image disturbance, compared to guided mirror exposure and imagery exposure.
Onden‐Lim and Grisham (2012)	*N* = 92 Mean age: 19.92 Sex: *M* = 25, *F* = 67 Diagnosis: No	Undergraduate students selected for elevated body dissatisfaction (BICI ≥ 56)	Imagery suppression vs. Imagery monitoring	BICI (Littleton et al. 2005), BDDDM	Self‐report forms; mood, discomfort, effort to suppress intrusions, vividness, believability and perspective of imagery.	Dysmorphic concern predicted the vividness and discomfort of appearance‐related intrusions but not frequency or suppression efforts. Suppression reduced frequency and discomfort temporarily but not vividness.
Osman et al. (2004)^c^	*N* = 36 Mean age: 27.5 Sex: *M* = 18, *F* = 18 Diagnosis: Yes	BDD patients and staff (controls) recruited in a UK‐based hospital	BDD vs. non‐clinical control group	BDD‐YBOCS, SCID‐IV and BCQ (Miller et al. 1981)	Semi‐structured interview; Participants were asked to recreate and describe their spontaneous images, including various sensory modalities. Idiographic scales measuring the characteristics of imagery.	BDD patients experienced significantly more frequent, vivid and negative mental images of their bodies compared to controls. BDD patients' images were more likely to be viewed from an external observer's perspective, reinforcing negative self‐appraisals. Visual imagery the most common with some other modalities present.
Pascual‐Vera et al. (2019)	*N* = 438 Mean age: Undergraduate = 24.37, Community = 40.86 Sex: *M* = 130, *F* = 308 Diagnosis: No	General population with no clinical diagnosis of BDD or body image disorder	N/A: This study was a cross‐sectional survey without any treatment or comparison groups.	None.	QUIT; presence, intensity and intrusiveness of BDD‐, OCD‐, health anxiety‐ and eating disorder‐related imagery	Occurrence of BDD‐related intrusions in clinical samples was high, often co‐occurring with OCD, eating disorders and health anxiety, and these intrusions were equally distressing regardless of content.
Pascual‐Vera et al. (2022)	*N* = 611 Mean age: 25.88 Sex: *M* = 122, *F* = 489 Diagnosis: No	International sample without recent mental health diagnoses; selected for having experienced upsetting unwanted mental images	N/A: This was a descriptive study exploring cultural and socioeconomic influences on appearance‐related intrusions. There were no treatment or clinical comparison groups.	None.	QUIT; presence, intensity and intrusiveness of BDD‐, OCD‐, health anxiety‐ and eating disorder‐related imagery	Unwanted mental intrusions (UMIs) related to OCD, BDD and eating disorders were experienced universally across different cultures.
Van den Berg and Thompson (2007)	*N* = 227 Mean age: 20.47 Sex: *M* = 0, *F* = 227 Diagnosis: No	Non‐clinical undergraduate at USA‐based university	Appearance schema priming vs. non‐appearance schema priming	None.	None used as outcome measures. Guided mental imagery was used to evoke appearance‐related comparisons to self‐relevant images.	Using guided imagery to experimentally induce appearance comparisons, downward social comparisons (viewing less attractive individuals) resulted in higher body satisfaction and self‐confidence compared to upward comparisons.
Veale et al. (2015)	*N* = 90 Mean age: BDD = 42.02, SPA = 31.77, Control = 31.55 Sex: *M* = 90, *F* = 0 Diagnosis: Yes	Adult men from community sampling; excluded men with penile abnormalities to control for visible difference.	BDD vs. Subclinical Penis Anxiety (SPA) vs. no concern with penis	BIQoLI (Cash and Fleming 2002), BAPS (Veale, Eshkevari, Read, et al. 2014); COPS‐P (Veale et al. 2012)	Idiographic; presence, distortion, recurrence and intrusiveness of self‐relevant penis imagery.	More participants with BDD reported experiencing recurrent imagery related to their penis compared to control groups. These images fell into categories such as flashbacks, flash‐forwards and distorted self‐images.
Veale et al. (2016)	*N* = 173 Mean age: 23.45 Sex: *M* = 0, *F* = 173 Diagnosis: No	Convenience sample of university staff and students, unselected for BDD or dissatisfaction	Self‐focus during mirror exposure vs. external focus of attention during mirror exposure	AAI (Veale, Eshkevari, Kanakam, et al. 2014; Yurtsever et al. 2022), MDBSRQ—Appearance Scales	MG‐CARS; affect and thoughts following self‐relevant images, additionally measured whether attention was paid to internal or external images	The study provides support for the use of imagery as a technique in mirror‐gazing exercises, although it primarily applies to practical applications rather than contributing new theoretical insights.

Abbreviations: AAI = Appearance Anxiety Inventory; AIQ = Appearance Intrusions Questionnaire; BAPS = Beliefs about Penis Size; BCQ = Body Consciousness Questionnaire; BDDDM = Body Dysmorphic Disorder Diagnostic Module; BDDQ = Body Dysmorphic Disorder Questionnaire; BICI = Body Image Concern Inventory; BIDQ = Body Image Disturbance Questionnaire; BIQoLI = Body Image Quality of Life Inventory; BSQ = Body Shape Questionnaire; COPS‐P = Cosmetic Procedure Screening Scale for BDD related to penile appearance; EAT = Eating Attitudes Test; EDI‐2 = Eating Disorder Inventory—2; IVIS = Intrusive Visual Imagery Scale; MDBSRQ = Multidimensional Body‐Self Relations Questionnaire; MG‐CARS = Mirror‐gazing: Cognition and Affect Rating Scale; N/A = Not applicable; OCD = Obsessive Compulsive Disorder; QUIT = Questionnaire of Unpleasant Intrusive Thoughts; SCID‐IV = Structured Clinical Interview for DSM‐IV; SDBPS = Satisfaction and Dissatisfaction with Body Parts Scale; VAS = Visual analogue scales.

^a^ This criterion was satisfied if the study includes any participants formally diagnosed with BDD. Studies passing this criterion may also contain clinical and non‐clinical control participants.

^b^ The main findings relevant to mental imagery in BDD: These may not reflect the main findings of the study overall.

^c^ A mixed methods study wherein the table presents only the quantitative findings.

**TABLE 3:** Summary of intervention studies included in quantitative synthesis.

Authors and years of publication	Population (*N*, age, sex and formal diagnosis^a^ Y/N)	Sample description	Intervention	Comparator(s) if applicable	BDD measure(s)	Imagery measure(s); aspects of imagery measured	Imagery procedure	Main findings^b^
Brown et al. (1997)	*N* = 7 Age range: Unspecified, teenage to forties Sex: *M* = 2, *F* = 5 Diagnosis: Yes	Case‐series of BDD presenting to mental health services, all with identifiable onset events and intrusive appearance‐related imagery (except one). Severe and chronic presentations.	Eye movement reprocessing and desensitisation (EMDR)	No comparison group.	Not reported.	No measures; sensory modality, intrusiveness, recurrence and emotional salience	Procedure not described in article.	EMDR was effective in cases where visual mental imagery played a significant role in the disorder, although one patient, who could not engage with imagery, did not respond to treatment.
Dziegielewski and Wolfe (2000)	*N* = 1 Age: 26 Sex: *M* = 0, *F* = 1 Diagnosis: No	White female, normal range BMI, familiar with EMDR	EMDR	No comparison group.	BIAQ (Rosen et al. 1991), Body Dissatisfaction Log	No measures; imagery of an appearance‐related event, cognitive‐emotional links of the imagery	Identified negative images in assessment stage, cognitive restructuring of the images and emotions in later stages.	EMDR could be a brief and effective intervention for improving self‐esteem and reducing body image disturbance in similar cases.
Ghaderi et al. (2022)—Study 2	*N* = 42 Age: Not reported, (range 18–45) Sex: *M* = 0, *F* = 42 Diagnosis: No	General population, with subjective sense of body dissatisfaction	Imagery rescripting (ImRs)	Expressive writing (as an active placebo); no intervention	BSQ and IBSS (Czepczor‐Bernat et al. 2017)	Visual Analogue Scales (VAS) for frequency, vividness, believability, controllability and distress.	Participants recalled the most intrusive mental image and described its characteristics, triggers and appraisals. Participants were guided to find an ‘antidote’ to the negative emotion and associated meaning.	ImRs and Expressive Writing (EW) reduced the vividness, believability, negative affect and distress associated with negative body‐related images.
Ghaderi et al. (2022)—Study 3	*N* = 113 Mean age: ImRs = 33.8, Expressive writing = 32.5, Waitlist = 31.9 Sex: *M* = 0, *F* = 113 Diagnosis: No	Community sample, self‐reported body dissatisfaction	ImRs	Expressive writing (as an active placebo); no intervention	BSQ and IBSS	VAS for frequency, vividness, believability, controllability and distress.	See Ghaderi et al. (2022) – Study 2	Both ImRs and Expressive Writing (EW) reduced body dissatisfaction and eating disorder symptoms.
Gu and Zhu (2023)	*N* = 116 Mean age: 32.5 Sex: *M* =, *F* = Diagnosis: Yes	All BDD diagnosed, recruited from clinical settings; all had moderate‐to‐severe BDD symptoms and physically healthy	Mindfulness‐based cognitive therapy (MBCT)	Treatment as usual (Unspecified pharmacological and psychosocial treatments)	BDD‐YBOCS, BABP and COPS	No measures; awareness of mental events including appearance‐related imagery	Body sensations and images evoked during mindfulness, patient encouraged to separate associated emotions. Authors described this as ‘imagery mediation’.	MBCT is feasible and acceptable for BDD patients, with participants reporting a trend towards reduced BDD symptoms.
McKay et al. (1997)	*N* = 10 Mean age: 31.2 Sex: *M* = 4, *F* = 6 Age range: 21–45 Diagnosis: Yes	Adults with BDD recruited from clinical settings, physically healthy	Exposure and response prevention (ERP)	No intervention	BDD‐YBOCS	No measures; imagery‐based exposure exercises to imperfections and emotional responses	Patient imagined a deformity in an exaggerated manner, providing exposure to threatening stimuli	ERP is efficacious in treating BDD symptoms.
Pouladi et al. (2024)	*N* = 4 Mean age: 27.5 Sex: *M* = 0, *F* = 4 Diagnosis: Yes	Adults with BDD diagnosis, recruited from psychotherapy clinics	EMDR	No comparison group.	BDD‐YBOCS and BISS (Thompson et al. 2003)	No measures; content of appearance‐related memories	In desensitisation stage, visual image of memories imagined and bilateral stimulation to disseminate this from associated thoughts and emotions	EMDR reduced BDD symptoms, appearance‐based rejection sensitivity and body shame, while increasing self‐compassion in BDD patients.
Rabiei et al. (2012)	*N* = 20 Mean age: 25.2 Sex: *M* = 2, *F* = 18 Diagnosis: Yes	Adults with BDD diagnosis, recruited at dermatology and cosmetic clinics in Iran	Metacognitive therapy	No intervention	BDD‐YBOCS and SCID‐IV‐TR	TFI; cognitive‐emotional linkage of imagery	Using ‘detached mindfulness’ to separate self from thoughts and reconsider their meaning	MCT reduced thought‐fusion symptoms in BDD, with large effect sizes observed post‐treatment.
Ritter and Stangier (2016)	*N* = 6 Mean age: 29.5 Sex: *M* = 1, *F* = 5 Diagnosis: Yes	BDD outpatients at a German university clinic, no comorbid PTSD, all with intrusive mental images about facial features	ImRs	No comparison group.	BDD‐YBOCS, SCID‐IV, BDDDM and BDSI	Semi‐structured interview adapted from previous studies. Single‐item scales for distress, vividness, controllability and encapsulated beliefs (scale: 0–100).	Imagining distressing past scenario and introducing current self to provide new meaning and information to the memory.	ImRs could be effective for treating BDD. The ability to recall and create vivid imagery seemed to be a critical factor in determining the success of the therapy.
Sündermann et al. (2016)	*N* = 1 Age: 25 Sex: *M* = 1 Diagnosis: Yes	Case study of male with severe, treatment‐refractory BDD in a UK‐based clinic	CBT with ImRs	No comparison group.	BDD‐YBOCS, AAI and BDD‐D (LeBeau et al. 2013).	No measures; content and emotional salience of memory	Changing the outcome of a previous memory of sexual abuse, to ‘rescue’ the former self	This case study showed the successful treatment of a young man with treatment‐resistant BDD using CBT enhanced with emotion‐focused techniques like ImRs and compassion‐focused therapy.
Veale, Anson, et al. (2014)	*N* = 46 Median age: 30 Sex: *M* = 19, *F* = 27 Diagnosis: Yes	Adults with BDD or high symptom severity (BDD‐YBOCS≥ 24) at UK outpatient clinic	CBT with ImRs	Anxiety management	BDD‐YBOCS and BABS (Eisen et al. 1998), AAI, BIQoLI	No measure; rescripting of cognitive‐emotional links of memory	No description other than ‘Imagery rescripting followed for past aversive memories that were associated with the onset’	CBT with ImRs was superior to Anxiety Management in treating BDD.
Willson et al. (2016)	*N* = 6 Mean age: 25.67 Sex: *M* = 2, *F* = 4 Diagnosis: Yes	Adults with BDD or moderate/high symptom severity (BDD‐YBOCS≥ 20), at UK outpatient clinic	ImRs	Describing imagery without intervention	BDD‐YBOCS and self‐report monitoring of BDD‐related preoccupation	No measures; engagement with memory image	Re‐entering a past scenario to alter the events in the memory, e.g., offering a hug to the past self.	ImRs improved preoccupation and distress, with changes sustained up to 6 months. The vividness of imagery seemed to influence effectiveness.

Abbreviations: AAI = Appearance Anxiety Inventory; BABS = Brown Assessment of Beliefs Scale; BDD‐D = Body Dysmorphic Disorder Dimensional Scale; BDD‐YBOCS = BDD‐Yale Brown Obsessive Compulsive Scale; BDDDM = Body Dysmorphic Disorder Diagnostic Module; BDSI = Body Dysmorphic Symptoms Inventory; BIAQ = Body Image Avoidance Questionnaire; BIQoLI = Body Image Quality of Life Inventory; BISS = Body Image Shame Scale; BSQ = Body Shape Questionnaire; CBT = Cognitive behavioural therapy; COPS = Cosmetic Procedure Screening Questionnaire; EMDR = eye movement desensitisation and reprocessing; ERP = Exposure and response prevention; IBSS = Ideal Body Stereotype Scale‐Revised; ImRS = Imagery rescripting; SCID‐IV‐TR = Structured Clinical Interview for DSM‐IV—Text Revision; VAS = Visual analogue scales.

^a^ This criterion was satisfied if the study includes any participants formally diagnosed with BDD. Studies passing this criterion may also contain clinical and non‐clinical control participants.

^b^ The main findings relevant to mental imagery in BDD. These may not reflect the main findings of the study overall.

#### 3.2.1 Characteristics of Mental Imagery

##### 3.2.1.1 Content

Most studies showed that imagery often exaggerates participants' perceived body flaws, particularly facial features including the nose, skin, eyes and lips (Grocholewski et al. 2018; Pascual‐Vera et al. 2019; Veale and Riley 2001). Scalp, hair, facial hair and body hair were also common focuses (Ghaderi et al. 2022), as were overall body shape concerns such as fat distribution, muscle tone and size (Giraldo‐O'Meara and Belloch 2018; Pascual‐Vera et al. 2019). Other areas of concern included teeth, limbs and genitalia (Veale et al. 2015; Willson et al. 2016). In younger adults (18–35 years), facial features such as the nose, eyes and skin were most common (Veale, Eshkevari, Kanakam, et al. 2014; Pascual‐Vera et al. 2022, 2019), while middle‐aged adults (35–65 years) more frequently reported concerns about hair thinning and body shape changes, likely reflecting age‐related concerns (McKay et al. 1997; Ritter and Stangier 2016). Gender differences were evident: males focused on muscle tone, hair thinning and genitalia (Ritter and Stangier 2016; Veale et al. 2016; Brown et al. 1997), while females focused on breasts and genitalia (Willson et al. 2016; McKay et al. 1997). Women more frequently focused on multiple body parts compared to men (Ritter and Stangier 2016; Giraldo‐O'Meara and Belloch 2018).

Imagery also often involved individuals comparing their distorted self‐images to a version of themselves they perceive as ‘normal’ or that others will deem acceptable (Willson et al. 2016; Veale, Anson, et al. 2014), contributing to body dissatisfaction (Onden‐Lim and Grisham 2012). A cross‐cultural study across Israel, Turkey, Iran, Italy, Portugal, Spain and Argentina found no major differences in imagery content, with slight variations in imagery reflecting local attitudes towards gender, appearance, religion and migration being contributable to age‐related differences rather than cultural differences (Pascual‐Vera et al. 2019).

##### 3.2.1.2 Intensity

Participants with a BDD diagnosis or high BDD symptomology consistently reported more intense (e.g., frequent, vivid, intrusive, emotionally distressing) body‐related mental imagery compared to non‐clinical groups (Pascual‐Vera 2019; Chuah and Suendermann 2024). In non‐clinical samples, although lifetime prevalence of at least one intrusive appearance‐related image is high (91.9%; Giraldo‐O'Meara and Belloch 2019), intensity was consistently lower than BDD samples (Giraldo‐O'Meara and Belloch 2018, 2019). The intensity of imagery appeared to be higher in participants of younger age, which may reflect increased BDD severity with younger ages in the relevant studies (Pascual‐Vera et al. 2019; Chuah and Suendermann 2024). Most studies reported men and women to experience imagery to a similar intensity (Chuah and Suendermann 2024), with the exception of one study (Giraldo‐O'Meara and Belloch 2019) that reported women to experience higher intensity.

##### 3.2.1.3 Generation

Triggers for intrusive imagery appear to include factors that activate negative appraisals about body image, such as anticipating a social event (Willson et al. 2016), mirror‐gazing (Veale et al. 2016) and social comparisons (Liu et al. 2023). Intrusive imagery was more frequent during heightened emotional arousal, especially anxiety (Onden‐Lim and Grisham 2012; Farrar et al. 2015). In some circumstances, such images can also be deliberately evoked, under the individual's volition and researcher/clinician's guidance, for example as part of imagery‐based interventive techniques, including exposure (McKay et al. 1997), ImRs (Willson et al. 2016), in vivo replication of body‐focused behaviours (e.g., mirror‐gazing; Veale et al. 2016) or body image rumination (Hamamoto et al. 2024).

##### 3.2.1.4 Sensory Modality

Imagery was overwhelmingly described in the visual domain, with only one (Osman et al. 2004) reporting auditory, internal and tactile imagery. This appears predominantly due to studies only measuring the visual domain (Moreno‐Domínguez et al. 2012; Farrar et al. 2015) and outcome measures only assessing visual imagery (Pascual‐Vera et al. 2019; Giraldo‐O'Meara and Belloch 2019), biasing the data towards visual domains.

##### 3.2.1.5 Emotional Impact

Mental imagery was consistently reported as negative in emotional tone (Veale, Eshkevari, Read, et al. 2014; Sündermann et al. 2016; Pascual‐Vera et al. 2019). Experiencing imagery further increased anxiety (Pascual‐Vera et al. 2022; Van Den Berg and Thompson 2007), depressive symptoms (Farrar et al. 2015; Veale et al. 2016; Hamamoto et al. 2024), shame (Brown et al. 1997) and body dissatisfaction (Hamamoto et al. 2024; Liu et al. 2023; Moreno‐Domínguez et al. 2012). While many studies describing these images naturalistically were cross‐sectional, experimental studies inducing negative imagery provided additional support for its causal role on emotional impact relevant to BDD.

##### 3.2.1.6 Behavioural Response

The emotional impact prompted behavioural responses to imagery, such as rumination on imagery (Pascual‐Vera et al. 2019), attempts to suppress imagery (Onden‐Lim and Grisham 2012), among hallmark safety‐seeking behaviours of BDD (Veale et al. 1996; Veale 2004). Samples with BDD demonstrated more consistent use of safety‐seeking behaviours like mirror‐checking and camouflaging in response to imagery than non‐clinical samples (Veale et al. 2015; Ritter and Stangier 2016). Overall, such intrusive mental imagery appears to worsen daily functioning, especially in social interactions, occupational performance and self‐esteem, all of which are linked to increased BDD symptomatology (Sündermann et al. 2016; Rabiei et al. 2012). Although societal pressures and gender norms related to appearance were sometimes referred to (Giraldo‐O'Meara and Belloch 2018, 2019; Veale et al. 2015), no study directly measured gender norms or societal pressures. Some data suggest women more often use multiple coping strategies and were more likely to use thought control strategies such as distraction and neutralising thoughts (Giraldo O'Meara and Belloch 2018), whereas men more consistently used mirror checking, grooming and posturing (Veale et al. 2016). Relevant studies mostly focused on thoughts and behaviours in response to imagery, with a lack of research directly on idiographic (e.g., cultural) differences in imagery and responses.

#### 3.2.2 Interventions

A variety of imagery‐based interventions were identified, which could inform our mechanistic understanding of imagery in BDD (e.g., its maintenance and resolution), displayed in Table 4. These approaches were motivated by theoretical underpinnings about why each approach is thought to benefit BDD symptoms, suggesting hypotheses for imagery‐related mechanisms that maintained BDD in the first place. Nevertheless, no studies directly assessed or tested these mechanistic hypotheses and thus remain speculative. In addition to the lack of direct testing of mechanisms, most intervention studies were of variable but mostly lower quality.

**TABLE 4:** Overview of imagery‐based interventions identified for addressing BDD in included studies.

Intervention	Summary	Imagery work applied to BDD	Hypothesised imagery‐related mechanism	*N*, studies
Imagery rescripting (ImRs)—either stand alone or within cognitive‐behavioural therapy (CBT)	Changes negative meanings of imagery from aversive memories.	Engages with and alters distressing memories. E.g., memory of being bullied for facial defect is attached to the meaning that the person feels worthless. This is changed to a new meaning (e.g., they just wanted to talk to me) by changing the image content (e.g., it was said in a positive tone).	Autobiographical memories and their understood meanings maintain BDD symptoms. Changing the content of imagery therefore ameliorate symptomology.	5, Ghaderi et al. 2022; Ritter and Stangier 2016; Sündermann et al. 2016; Veale, Anson, et al. 2014; Willson et al. 2016
Exposure and response prevention (ERP)	Gradual exposure to feared stimuli without performing safety behaviours	Imagined generation of feared stimuli by simulation of feared scenarios (e.g., body being viewed by another) to reduce avoidance and distress (e.g., not posturing or looking away).	Avoidance of imagery maintains anxiety and negative interpretations related to the imagery content. Exposure without safety‐seeking behaviour interrupts this cycle.	1, McKay et al. 1997
Eye movement reprocessing and desensitisation (EMDR)	Processes trauma or stressful images using bilateral stimulation to reduce emotional distress	Imaginal generation of distressing memories and images related to BDD (e.g., memory of being bullied about appearance) while performing bilateral stimulations (e.g., performing horizontal eye movements while following the therapist's fingers).	Negative quality and appraisals of imagery are related to how they are processed (encoded, stored and retrieved). EMDR reprocesses the memory and desensitises the person to its content through imaginal generation and working memory competition.	3, Brown et al. 1997; Dziegielewski and Wolfe 2000; Pouladi et al. 2024
Metacognitive therapy (MCT)	Targets dysfunctional beliefs about own thinking processes	Examines and targets negative beliefs (e.g., I am worthless because I am unattractive) about the experience of imagery (e.g., if I keep thinking about how unattractive I am, I might find a solution).	Beliefs about engaging in imagery processes are associated to negative qualities of imagery and the perpetuation of safety‐seeking behaviours. Changing beliefs about imagery may disrupt this process.	1, Rabiei et al. 2012
Mindfulness based cognitive therapy (MBCT)	Uses cognitive defusion and mindfulness techniques to help clients disentangle mental imagery from its perceived meaning	The client is encouraged to engage with mental imagery rather than avoid it (e.g., be aware of distorted body parts) and recognise this as an internal experience rather than a reflection of reality	Cognitive fusion between mental imagery and its perceived meaning perpetuates BDD symptoms; mindfulness‐based techniques promote decoupling of imagery from meaning	1, Gu and Zhu 2023

##### 3.2.2.1 ImRs

The three studies using ImRs as a standalone intervention were generally high quality and demonstrated efficacy in reducing imagery vividness, emotional impact and improving BDD symptoms (Ghaderi et al. 2022; Ritter and Stangier 2016; Willson et al. 2016). Similarly, two studies highlighted CBT with ImRs as effective in reducing BDD symptoms (Sündermann et al. 2016; Veale, Anson, et al. 2014). Mechanistically, these studies consistently suggested early traumatic/negative appearance‐related experiences led to associations between negative appraisals of body image and body‐related experiences. ImRs addresses this by targeting the distressing memories underpinning BDD symptoms, allowing for a rescripting of the memory to reduce its emotional impact. Unlike standard CBT, which primarily focuses on verbal cognitive distortions, ImRs leverages the emotional salience of mental imagery, the encapsulated meaning within its aversive memory of origin and reducing the perceived immediacy of memories as key mechanisms of change. Although one single case experiment reported significant changes in strength of beliefs from imagery rescripting (Willson et al. 2016), changes in cognitive beliefs resulting from imagery rescripting were not consistently assessed.

##### 3.2.2.2 ERP

One study using ERP, which was poor in quality, demonstrated the use of imaginal exposure to perceived physical flaws as a way of reducing negative emotional responses to one's body and facilitating the prevention of safety‐seeking behaviours (McKay et al. 1997). At the time of this publication, this approach was grounded in traditional exposure models, which proposed that repeatedly confronting feared stimuli, whether real or imagined, would lead to habituation and a reduction in distress over time. While the study did not explicitly frame its findings in terms of inhibitory learning, this later theoretical development suggests that imaginal exposure may also function by weakening threat associations and increasing distress tolerance (Craske et al. 2014). ERP activates negative emotions and beliefs associated with body image, using inhibitory learning principles to allow extinction of associated beliefs, tolerance of distress and opportunity to test assumptions about responses to distressing stimuli through repeated exposure without engaging in safety‐seeking behaviours. While this study scored as ‘poor’ on quality from significant underreporting of findings, it demonstrated an early approach to applying principles of imagery exposure for BDD, drawing from related work with anxiety disorders (Ferrando and Selai 2021).

##### 3.2.2.3 EMDR

Three studies suggest EMDR can be effective for BDD symptoms, through focusing on reprocessing of past memories and their relationship to future‐focused images associated with one's perceived flaws, in conjunction with bilateral simulations (Brown et al. 1997; Dziegielewski and Wolfe 2000; Pouladi et al. 2024). The three studies were mixed in quality, ranging from good to poor. While Pouladi et al. (2024) reported a 60.5% reduction in BDD symptoms, the other studies did not quantify change in BDD, only claiming subjectively that the interventions were effective. Mechanistically, experimental work has proposed that EMDR operates by reprocessing distressing memories and disrupting working memory involved in imagery generation, rendering the images less vivid and emotional for restorage in long‐term memory (Van den Hout and Engelhard 2012). However, such mechanisms were not directly tested.

##### 3.2.2.4 MCT

The one study using MCT (Rabiei et al. 2012), which scored low in quality, reported reductions in BDD symptomology via an indirect approach to working with images. Rather than changing the image's content, as with previous approaches, a metacognitive approach aims to target the beliefs sustaining their preoccupation (e.g., ‘If I keep thinking about my appearance, I will find a solution’). This study offers preliminary evidence of using mental imagery to explore links between BDD severity, maladaptive beliefs and thinking processes and beliefs about imagery, although further investigations are needed to clarify the role of imagery in metacognitive approaches to BDD.

##### 3.2.2.5 MBCT

One study evaluated MBCT for BDD (Gu and Zhu 2023) compared to treatment‐as‐usual pharmacological and psychosocial interventions, demonstrating relative efficacy in reducing BDD symptoms. The study scored highly in quality. This could be considered an indirect approach for working with imagery, similar to MCT. Rather than changing image content (whether these are mental images or external reflections such as in mirror‐gazing), MBCT involved responding to spontaneous images that emerge using techniques such as present‐moment awareness and decentring which aim to increase a focus on current (e.g., sensory) experiences and decrease evaluative self‐referential processing. The study suggests that how we respond to images (e.g., the use of certain unhelpful emotional regulation strategies such as avoidance or suppression) may contribute to negative self‐referential processing. Further research is needed to clarify MBCT's specific role in modifying imagery‐related distress in BDD.

### 3.3 Qualitative Synthesis

Eight studies were included in the qualitative synthesis (see Table 5). Studies focused broadly on the presence and nature of intrusive imagery (Osman et al. 2004; Ghaderi et al. 2022; Onden‐Lim and Grisham 2012), metacognition (Cooper and Osman 2007), self‐identified origins and experiences of BDD (Craythorne et al. 2022; Brennan et al. 2023), perceptions about their younger selves (Silver and Reavey 2010) and physically intimate relationships (Stechler and Henton 2022). There were no qualitative studies which spoke to individuals' experiences of imagery‐based interventions or perceived mechanisms of change.

**TABLE 5:** Summary of studies included in the qualitative synthesis.

Authors	Population (*N*, age, sex and formal diagnosis^a^ Y/N)	Sample descriptors, clinical group and/or comparison groups, (if applicable)	BDD measure(s)	Imagery measure(s) and aspects of imagery measured	Main findings^b^
Cooper and Osman (2007)	*N* = 18 Mean age: 27.5 Sex: *M* = 9, *F* = 9 Diagnosis: Yes	BDD participants recruited from a specialist clinician based in the United Kingdom. No group comparisons.	SCID‐IV	Semi‐structured interview. Participants identified an image related to concerns about appearance, described it in detail and answered questions about metacognition.	BDD patients frequently experienced negative body‐related images, leading to negative self‐judgements, with many relying on coping strategies such as distraction and avoidance. Images were often linked to early memories and mirror‐gazing, reinforcing negative appraisals of their appearance.
Craythorne et al. (2022)	*N* = 8 Mean age: 32.13 Sex: *M* = 2, *F* = 6 Diagnosis: No (Some participants were in the process of getting a diagnosis)	Self‐identified BDD participants, located in the United Kingdom, United States and India. No group comparisons.	BDDQ	None. Mental imagery emerged as a theme from semi‐structured interviews about self‐identified origins and experiences of BDD.	Participants reported experiencing negative self‐appraisals that were linked to appearance‐focused bullying from a young age. The imagery associated with these experiences led to ongoing self‐criticism and negative perceptions of their appearance, reinforcing feelings of shame and distress.
Ghaderi et al. (2022)‐ Study 1^c^	*N* = 31 Mean age: 27.8 Sex: *M* = 0, *F* = 31 Diagnosis: No	Individuals high in body dissatisfaction based in Sweden. No group comparisons.	BSQ and IBSS	Semi‐structured interview on content of mental imagery.	Imagery can be positive or negative in emotional tone, but especially negative in the context of greater body dissatisfaction.
Onden‐Lim and Grisham (2013)	*N* = 65 Mean age: 19.32 Sex: *M* = 10, *F* = 55 Diagnosis: No	Undergraduate students in Australia with high dysmorphic concern (BICI≥ 56). No group comparisons. Range of ethnicities (38% Anglo‐Saxon, 35% Asian, 20% mixed/other)	BICI	Semi‐structured interview adapted from Osman et al. (2004). Participants described their most frequent recurrent image and answered questions about the content, context and quality.	Participants reported frequent intrusive imagery related to body concerns, with negative imagery often reflecting social situations or self‐comparisons. Attempts to suppress these images were more common in BDD, but no direct link to image frequency or vividness was observed.
Osman et al. (2004)^c^	*N* = 36 Mean age: 27.5 Sex: *M* = 18, *F* = 18 Diagnosis: Yes	BDD patients and staff (controls) recruited in a UK‐based hospital	BDD‐YBOCS, SCID‐IV and BCQ	Semi‐structured interview. Participants were asked to recreate and describe their spontaneous images, including various sensory modalities. Idiographic scales measuring the characteristics of imagery.	Images in BDD were often linked to stressful early memories, including bullying. The images may be reconstructions of the memories themselves or novel generated images around the same themes as the early stressful memories.
Silver and Reavey (2010)	*N* = 11 Mean age: Not reported Age range: 20–39 Sex: *M* = 7, *F* = 4 Diagnosis: Yes	Self‐reported BDD participants in the United Kingdom, recruited through an OCD clinic and a self‐help group. No group comparisons.	Self‐report diagnosis only	Semi‐structured interview. Participants were asked to bring photographs to prompt imagery and memories; discussed photographs and drew a picture of themselves.	Individuals view their past selves in idealistic manners and lament the loss of that appearance and meanings attached to more youthful appearance, demonstrating a role of self‐objectification in the negative appraisals associated with BDD.
Stechler and Henton (2022)	*N* = 6 Mean age: Not reported Age range: 21–33 Sex: *M* = 0, *F* = 6 Diagnosis: Yes	Female adults in heterosexual relationships, most (4/6) had BDD diagnosis. Recruited through BDD and OCD charities. No group comparisons.	Self‐identification with BDD and self‐report diagnosis of BDD	None. Mental imagery emerged as a theme from semi‐structured interviews about phenomena of BDD and physical intimacy.	Participants reported using their partners' imagined view of their bodies to critically appraise their appearance, leading to distress. Negative comparisons with idealised images of other women contributed to dissociative experiences during intimate moments, further disrupting intimate experiences.
Brennan et al. (2023)	*N* = 12 Mean age: Not reported Age range: 19–64 Sex: *M* = 5, *F* = 7 Diagnosis: Yes	BDD diagnosed participants from a UK‐based specialist BDD service. No group comparisons.	BDD‐YBOCS, BDDDM and MINI	None. Mental imagery emerged as a theme from semi‐structured interviews about lived experience of BDD.	Individuals with BDD frequently experience distressing mental imagery related to their body, often on specific body parts like skin, hair and facial features. Images were often seen from an observer's perspective, contributing to feelings of objectification and hyper‐awareness in public.

Abbreviations: BCQ = Body Consciousness Questionnaire; BDDQ = Body Dysmorphic Disorder Questionnaire; BDD‐YBOCS = BDD‐Yale Brown Obsessive Compulsive Scale; BICI = Body Image Concern Inventory; BSQ = Body Shape Questionnaire; IBSS = Ideal Body Stereotype Scale‐Revised; MINI = Mini‐International Neuropsychiatric Interview; OCD = Obsessive Compulsive Disorder; SCID‐IV = Structured Clinical Interview for DSM‐IV.

^a^ This criterion was satisfied if the study includes any participants formally diagnosed with BDD. Studies passing this criterion may also contain clinical and non‐clinical control participants.

^b^ The main findings relevant to mental imagery in BDD. These may not reflect the main findings of the study overall.

^c^ A mixed methods study wherein the table presents only the qualitative findings.

**FIGURE 4:**
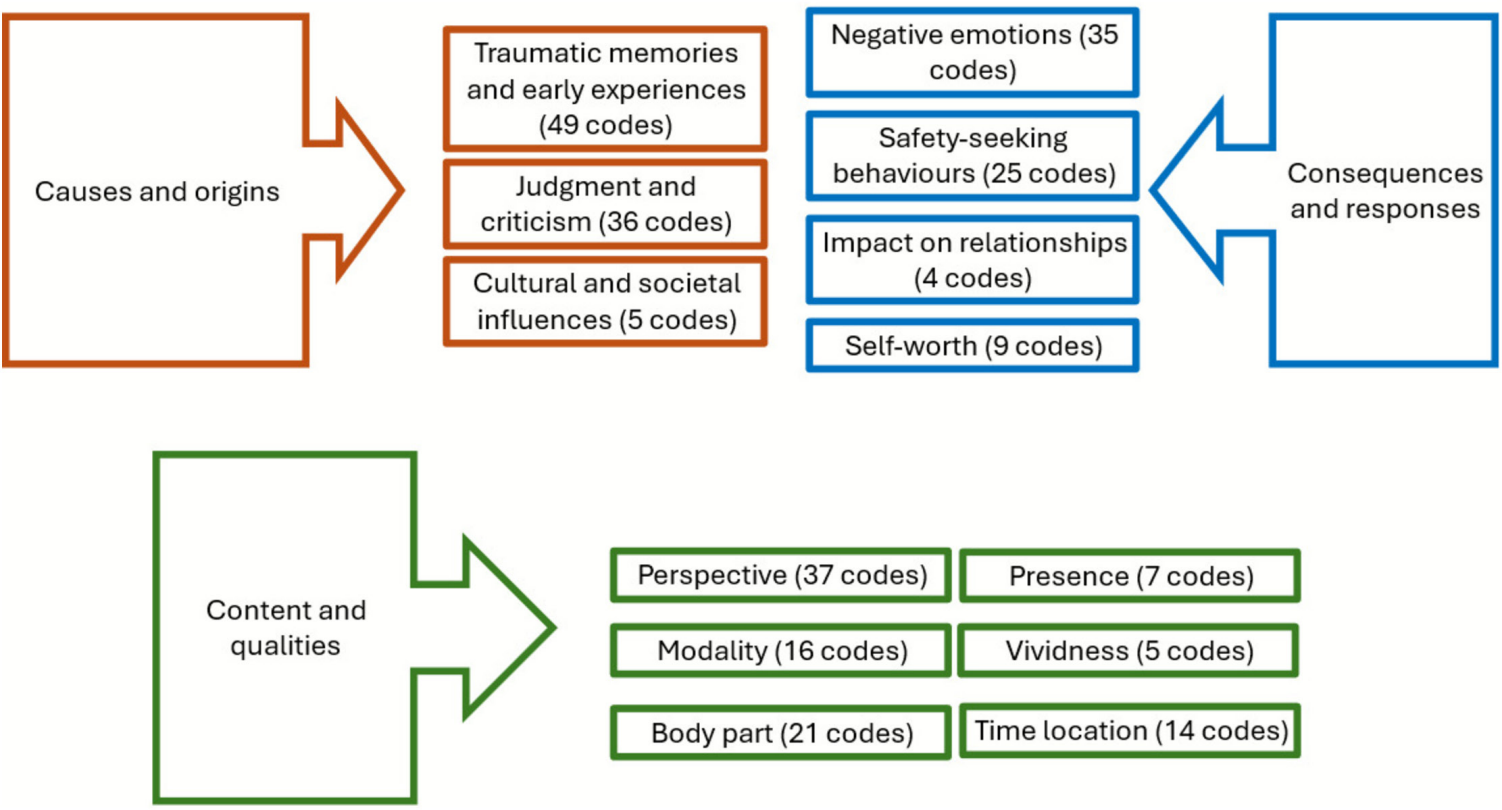
Analytical and descriptive themes from thematic synthesis.

Thematic synthesis led to the generation of three analytical themes: (1) causes and origins; (2) content and qualities; (3) consequences and responses, shown graphically in Figure 4. These were derived from 263 initial codes and 13 descriptive themes, detailed in the Supporting Information.

#### 3.3.1 Causes and Origins

Early life experiences, particularly involving appearance‐related criticism or comparison, were often involved in the onset of BDD in those who experience intrusive imagery (Craythorne et al. 2022; Osman et al. 2004). Common elements included bullying, teasing or negative comments from peers, family or other influential figures, for example: ‘I do remember in detail how he teased me … it is vivid in my mind during and after’ (Craythorne et al. 2022). These experiences typically occurred between ages 7 and 15 (Craythorne et al. 2022; Silver and Reavey 2010), which coincided with adolescence, when self‐consciousness around appearance was highest (Brennan et al. 2023). Participants regularly expressed how cultural and familial pressures around appearance were significant throughout childhood, with a pervasive sense of appearance‐related judgement and criticism endemic to their upbringing: ‘I do not remember being appreciated or encouraged for anything by [family]. I remember getting shamed often, in comparison to other kids of my age, my cousins and friends’ (Craythorne et al. 2022).

#### 3.3.2 Content and Qualities

The modality of imagery in BDD was predominantly visual: two studies also found this could include auditory and tactile elements, while gustatory and olfactory experiences are rare (Osman et al. 2004; Onden‐Lim and Grisham 2013). Imagery was usually from a third‐person perspective (Brennan et al. 2023; Ghaderi et al. 2022), providing a sense of disconnection from one's body (Stechler and Henton 2022). These images often zoomed graphically on perceived flaws, most commonly skin, hair and facial features (Cooper and Osman 2007; Osman et al. 2004) in an exaggerated manner (Brennan et al. 2023). The emotional tone was overwhelmingly negative, frequently evoking shame, anxiety and disgust (Stechler and Henton 2022; Craythorne et al. 2022).

Imagery also involved self‐comparisons to ‘normal/acceptable’ perceptions of appearance, idealised versions of beauty (Stechler and Henton 2022) or scenarios where family members, peers or romantic partners were thought to be critically evaluating them (Stechler and Henton 2022; Cooper and Osman 2007). The imagery could take the form of static, snapshot images or slideshows and videos (Osman et al. 2004; Silver and Reavey 2010). Scenarios included specific memories, the current situation the person was in or purely imagined scenarios, which could be themselves in social and/or interpersonal settings or themselves in isolation (Silver and Reavey 2010; Onden‐Lim and Grisham 2013).

#### 3.3.3 Consequences and Responses

Imagery of memories and future‐oriented scenarios could be generated easily and vividly, evoking distress during interview (Brennan et al. 2023; Stechler and Henton 2022). The effect of significant past experiences continued to drive feelings of inadequacy and preoccupation with perceived flaws in the present day (Brennan et al. 2023; Onden‐Lim and Grisham 2013) and recurrent imagery prevented these experiences from reducing in emotional impact (Craythorne et al. 2022).

The negative emotional tone of imagery appeared to be due to activation of negative appearance‐related appraisals (Cooper and Osman 2007), which heightened attention to the images, in turn increasing their vividness and persistence. As more attention was paid to imagery, ruminative appearance‐related thoughts (e.g., ‘Do they think I'm ugly?’) increased, furthering attention and establishing a ruminative maintenance cycle (Stechler and Henton 2022; Craythorne et al. 2022).

Safety‐seeking actions such as hiding or camouflaging perceived flaws were common during and following imagery experiences. Individuals imagined what others saw using third‐person imagery, using this to guide their concealment efforts (Onden‐Lim and Grisham 2013; Brennan et al. 2023) or prompt avoidance from social situations (Stechler and Henton 2022).

As instances of withdrawal and avoidance accumulated and behavioural responses to imagery developed in sophistication, the disruption to daily functioning increased, interfering with concentration, social interactions or routine tasks (Cooper and Osman 2007; Brennan et al. 2023). Feelings of self‐judgement, hopelessness and emotional detachment were common, particularly when individuals felt trapped in cycles of negative self‐perception (Brennan et al. 2023). Over time, repeated instances of withdrawal reinforced negative appraisals, reducing self‐esteem, creating a sense of inadequacy and maintaining distressing cycles of imagery (Stechler and Henton 2022).

### 3.4 Meta‐Integration

**FIGURE 5:**
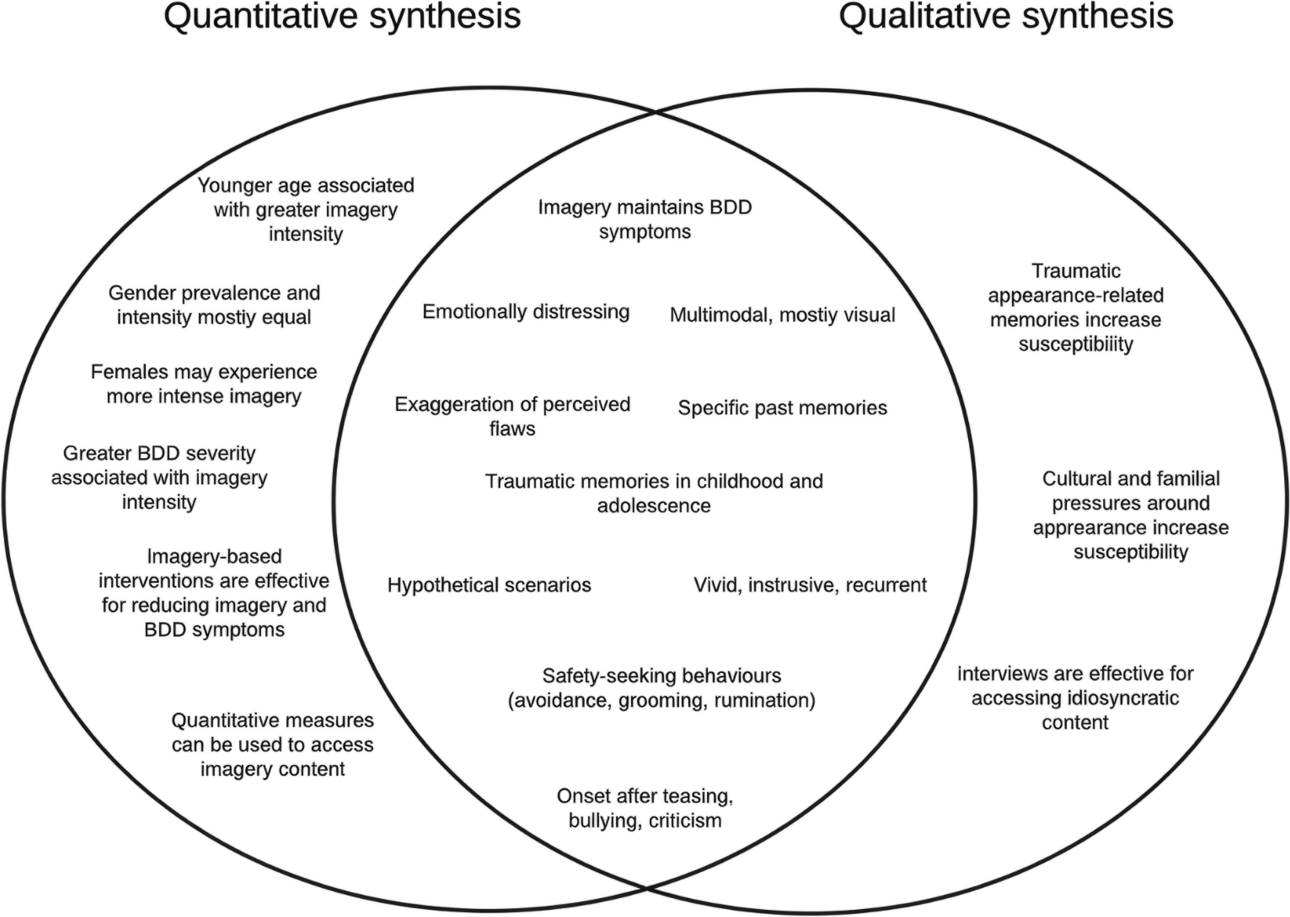
Graphical representation of the complements and unique contributions of both syntheses.

Overlapping and unique contributions of both syntheses, displayed graphically in Figure 5, were organised into characteristics and hypothesised mechanisms.

#### 3.4.1 Characteristics

Both datasets characterised mental imagery in BDD as emotionally distressing, vivid, intrusive and recurrent representations of distorted bodily perceptions, involving specific memories and hypothetical scenarios, most commonly from a third‐person perspective. Both datasets demonstrated the magnitude of these variables closely associating with emotional states like anxiety, shame and disgust, as well as maladaptive behaviours such as avoidance and mirror‐checking.

Both data sets complemented in imagery being primarily visual, with occasional instances of auditory, tactile and internal mental representations (Osman et al. 2004). As preoccupations in BDD primarily concern the aesthetic quality of perceived bodily flaws, it is perhaps unsurprising that visual imagery is most common in BDD. Yet, preoccupations may also regard bodily function and ability, which might feasibly be imagined more vividly in other modalities (e.g., touch and physical pain).

Individual differences were explored primarily within the quantitative dataset. Studies commenting on such differences generally demonstrated relatively high quality; but given the small number of studies this aspect synthesised remains tentative. The frequency, recurrence, vividness and intrusiveness of imagery appear to be positively associated with BDD severity and younger age. Ethnic and gender differences in BDD imagery appear to predominantly concern the specific content of imagery, often reflecting cultural or societal standards associated with these demographics, rather than the phenomenological qualities of imagery itself. In this sense, the images' content reflects motivational concerns of the individual in meeting certain socially approved, culturally informed aesthetic standards.

#### 3.4.2 Hypothesised Mechanisms

Quantitative and qualitative studies converge to suggest several mechanisms to explain a link between intrusive imagery and BDD. As few studies employed experimental or interventive designs to directly test mechanisms, these promising explanations require further empirical confirmation.

##### 3.4.2.1 Imagery as a Maintenance Factor in BDD

Both datasets highlighted an association between the intensity of imagery and maladaptive behaviour patterns which maintain BDD symptoms and further imagery. Intrusive imagery appeared to be triggered acutely by heightened negative emotions, especially anxiety, shame and disgust, anticipating social events and during social comparisons. Imagery activated negative self‐appraisals and self‐focused attention, furthering negative emotions and prompting behavioural responses.

##### 3.4.2.2 Imagery as a Potentially Vulnerability Factor for BDD

The quantitative dataset indicated greater intensity of imagery in BDD and high‐risk non‐clinical samples, suggesting imagery intensity exists on a continuum increasing with BDD severity (and thus strengthens our initial rationale for including non‐clinical body dissatisfaction in our review). Accordingly, several studies in both datasets (Giraldo‐O'Meara and Belloch 2018; Liu et al. 2023; Onden‐Lim and Grisham 2013) suggest that imagery is involved in the onset of BDD, not only its maintenance. Yet, as all relevant studies were retrospective and/or lacked control conditions, causal inferences are limited. It is possible that some individuals are more susceptible to maladaptive responses to vivid, emotional and recurrent imagery (of body‐related concerns) and less so to the adaptive functions of imagery (e.g., problem solving, positive self‐conceptualisation and appreciation of art), increasing susceptibility to BDD. Available data do not consider potential adaptive functions of (certain forms of) imagery in BDD, marking a notable knowledge gap for further clarification.

##### 3.4.2.3 Traumatic Early Experience as an Aetiological Risk Factor for Imagery

The qualitative synthesis suggests that particularly traumatic and/or impactful early experiences related to body image engender susceptibility to distressing intrusive imagery depicting these experiences (Section 3.3.2) as well as to BDD. Although previous reviews have proposed links between trauma history and BDD onset (Longobardi et al. 2022), it is not currently discernible if this association is explained by an increased susceptibility to distressing imagery following stressful events.

##### 3.4.2.4 Imagery as a Modifiable Treatment Target in BDD

The quantitative synthesis demonstrated imagery‐based interventions, either targeting imagery directly by modifying its content (e.g., ImRs) or indirectly by changing one's relationship to it (e.g., MCBT and MCT), effectively reduced imagery intensity and BDD symptoms. This is consistent with the notion that imagery is an important maintenance factor of BDD, although further research is needed to test this hypothesis. Accessing the content of imagery may elucidate the origin, nature and maintaining factors of one's BDD preoccupations (Stechler and Henton 2022; Silver and Reavey 2010) and provide means to more readily access and challenge negative appraisals (Ghaderi et al. 2022; Pouladi et al. 2024). As such, both qualitative and quantitative data indicate that a focus on imagery is relevant for developing formulation and guiding treatment in BDD. The mechanisms of change in imagery‐based interventions for BDD require further investigation.

## 4 Discussion

This review aimed to synthesise the evidence on the nature of self‐relevant, affect‐laden mental imagery in BDD and the mechanisms of imagery in BDD development, maintenance and amelioration. Our Meta‐Integration highlights imagery as a common, multimodal (though predominantly visual), recurrent and intrusive aspect of BDD symptomology. Imagery is typically of the self, viewed from a distorted, critical and third‐person perspective, which is consistent with the hallmark features of BDD, wherein the self or identity is strongly defined by perceived appearance defect(s). Image content often comprises specific early experiences or present/future hypothetical scenarios involving appearance‐related criticism. Though imagery can serve many adaptive functions in our mental lives (e.g., problem‐solving, creativity and self‐regulation; Kosslyn et al. 2006), within BDD symptomology the content appears to reflect perceived body flaws, negative appraisals about these flaws as well as societal and cultural meanings attached to flaws (Pascual‐Vera et al. 2022). Overall, these images capture key preoccupations relevant to a BDD presentation. As a first review on this topic in the field, we were deliberately inclusive in our scope and considered studies across a range of designs and populations to provide a comprehensive synthesis for applied and academic researchers interested in advancing a cognitive and clinical understanding of BDD but have little knowledge of imagery in BDD, such as phenomenological properties and potential interventions.

Imagery intensity (as captured e.g., by vividness, negative emotionality, intrusiveness and recurrence) was positively associated with higher BDD severity and with heightened emotional states, engendering self‐focused attention (Brennan et al. 2023; Osman et al. 2004). Although such imagery seems to exist on a continuum in the general population, it appeared far more common in those with high BDD symptomatology in both non‐clinical and clinical groups (Giraldo‐O'Meara and Belloch 2018). Similarly, imagery's intensity and associated responses (e.g., attention, interpretation and behavioural reactions) appeared to be more impairing in those with BDD. As such, intense intrusive mental imagery may be a somewhat distinguishing feature when comparing clinical and non‐clinical presentations of BDD. However, no clear‐cut boundary between normal and disorder can yet be discerned, at least purely based on subjective reports of intensity; thus, improved measurements are needed.

The available evidence complements cognitive‐behavioural frameworks of mental imagery in BDD, wherein self‐relevant imagery is maintained and intensified by negative body‐focused appraisals, negative emotions and behaviours intended to reduce distress (Cooper and Osman 2007). Imagery and other cognitive‐behavioural processes reinforce each other, maintaining BDD, consistent with models such as the ‘self as an aesthetic object’ framework (Veale 2004; Veale et al. 1996), which place centrality on imagery in the course of BDD.

Psychological interventions utilising imagery for BDD appear to be both feasible and effective (Ghaderi et al. 2022; Veale, Eshkevari, Kanakam, et al. 2014). Intervention studies already exist for BDD which incorporate addressing BDD‐related intrusive imagery directly (e.g., changing one's relationship and perceived immediacy of imagery via ImRs) or indirectly (e.g., changing metacognitive beliefs about those images via MBCT). The theoretical basis inspiring these interventions suggests multiple imagery‐related psychological mechanisms maintaining BDD that are targets for intervention, including negative appraisals, metacognitive beliefs (e.g., cognitive fusion), perceptual qualities of the image and safety‐seeking behavioural responses maintaining imagery intensity. Although the preliminary evidence of interventions' effectiveness is consistent with these proposed mechanisms, direct empirical testing within intervention designs has been limited. Notable exceptions include studies comparing imagery‐based interventions to control conditions that engage with but do not explicitly modify imagery (e.g., Ghaderi et al. 2022; Willson et al. 2016), demonstrating the potential for mechanistic testing within intervention research.

Further, intervention studies often measured imagery and BDD symptoms concurrently as outcome variables, limiting mechanistic claims, i.e., that changes in imagery precede changes in BDD symptoms. Future research with appropriate staggered timings of these measurements would strengthen claims that imagery serves as a mechanistic intervention target for BDD. Experimental studies with nonclinical populations would also be beneficial, given that our findings are consistent with the continuum perspective that imagery for body dissatisfaction is informative of BDD. Additional mechanisms at other levels of explanation (e.g., neural and social factors) may similarly benefit from further exploration.

In the meantime, the intervention and experimental studies reviewed highlight imagery as a modifiable cognitive target in BDD and patients find imagery‐focused interventions broadly accessible and feasible, in line with the established use of imagery‐based procedures primarily used for treating a range of anxiety disorders (Morina et al. 2017; Chapman et al. 2020). Therefore, therapeutics incorporating imagery are a fruitful area for further work and exploring possibilities for prevention and early interventions. For instance, while these techniques have demonstrated efficacy within broader therapeutic frameworks such as CBT and MBCT, further investigation is needed to determine whether they can be delivered effectively as standalone interventions or if additional cognitive‐behavioural components are necessary. Additionally, research could explore how best to combine and sequence various imagery‐based techniques, both direct and indirect ones, to maximise therapeutic outcomes. Given the potential influence of autobiographical memories in the development of BDD, it would be valuable to examine whether memory‐focused approaches (Dalgleish and Hitchcock 2023; Lau‐Zhu et al.  2023), particularly those incorporating imagery, can be adapted for this population. Moreover, principles of imagery disruption, similar to those employed in EMDR (Rackham and Lau‐Zhu 2021; Van den Hout and Engelhard 2012), may offer a basis for developing novel therapeutic approaches tailored to the unique needs of individuals with BDD.

Early experiences involving appearance‐related judgement and criticism can create vivid and intrusive imagery that persists into adulthood, acting as a core mechanism in the maintenance and potentially onset of BDD (Craythorne et al. 2022; Osman et al. 2004). The combination of these experiences and the distressing imagery they generate suggests a potential avenue for identifying individuals at risk of developing BDD. This aligns with contemporary frameworks in developmental psychopathology, which propose early adversity may give rise to latent cognitive vulnerabilities (McCrory and Viding 2015). While these vulnerabilities might not immediately manifest as psychopathology, they can increase the likelihood of its emergence in later stages. With further work, novel screening tools may be able to detect those with trauma histories and experiencing intrusive appearance‐related imagery, potentially allowing early intervention before BDD develops.

Adolescence, a period marked by heightened self‐consciousness and formation of self‐image (Phillips 2017), may be an ideal time to introduce preventative strategies. Despite the peak age of onset for BDD being in this period, intervention development targeted at this age remains underexplored (Longobardi et al. 2022). Previous work has highlighted the untapped potential of considering mental imagery in youth psychopathology (Heyes et al. 2013). Evidence is emerging that intrusive images are experienced in adolescence (Schwarz et al. 2020) and across developmental differences (Lau‐Zhu et al. 2024). Adolescents and young adults seem to find imagery work appealing for treating emotional difficulties across emotional disorders (e.g., Lau‐Zhu et al. 2022; Lau‐Zhu, Tuxen, et al. 2023), which could be relevant both for BDD.

The use of semi‐structured interviews focusing on imagery appears effective in elucidating key aspects of BDD psychopathology (Cooper and Osman 2007; Osman et al. 2004), beyond the content of imagery itself. Qualitative data demonstrated intimate links between imagery and the origins, course and maintaining factors of individuals' difficulties (Craythorne et al. 2022). Imagery‐based interviews can therefore be leveraged in the formulation and treatment planning stages of psychological interventions, while additional BDD‐relevant imagery measures can be developed/improved for informing screening and interventions.

### 4.1 Research Limitations and Future Directions

Significant methodological gaps were identified in the reviewed studies. Although the qualitative research and RCT interventions were typically of higher quality, most other studies suffered from unrepresentative samples for the BDD population, under‐justified use of measures and limited control for confounds. These issues were often driven by underreporting of studies' methods and results sections. While useful for early‐phase intervention development, several interventive studies were based on analogue samples, potentially limiting the applicability of their findings to BDD samples prior to formal evaluation. Participant recruitment ought to consider more representative samples and across the lifespan to better understand prevalence and generalisability, particularly in young people given the time window for BDD vulnerability and for early intervention.

Given the reliance on correlational or case–control designs, more robust evidence is needed of causal links of imagery in BDD disorder developmental/maintenance, such as using experimental and longitudinal approaches. Correspondingly, no studies to date have examined the moment‐to‐moment stability, persistence and pervasiveness of imagery content and responses (e.g., whether intensity fluctuates within the same day or across contexts). Mechanistic tests of imagery‐BDD links should be further explored, which could also be incorporated within intervention studies to better understand how imagery‐based approaches can be beneficial for BDD and who may respond better to such approaches. For example, clinical tools facilitating mechanistic exploration, such as clinical network analysis, have seen emerging application in a range of psychological disorders (e.g., van den Berg et al. 2024) and may supplement these knowledge gaps in BDD.

To better study individual differences, further psychometric validation/development of quantitative imagery measures specifically for BDD would be fruitful, as the field has mostly relied on semi‐structured imagery interviews without psychometric information, such as that employed by Osman et al. (2004), Onden‐Lim and Grisham (2012) and Grocholewski et al. (2018). Moreover, several instruments assessing imagery were not directly related to the mental imagery itself but its consequences (e.g., TFI, VOC and SUD). As most studies have also focused heavily on visual imagery, other sensory modalities (e.g., tactile and auditory) could be further explored in such measure development studies, in line with the reviewed qualitative data. Lastly, imagery content across studies was often more related to intrusive memories of past experiences (Iyadurai et al. 2019) than to representation of dysmorphic body parts—more research is needed to clarify potential differences between memory‐based imagery from other types within the BDD context.

Most studies focused on adults in their country of origin, with few drawing comparisons between demographics. This is especially relevant as the available evidence points to the origins, maintenance and content of imagery being largely idiosyncratic to one's developmental experience and the adolescent onset for BDD. Lived experience data of imagery in BDD from minoritised groups, such as ethnic minorities (relative to country of publication), the 0–18 and 65+ age groups and transgender populations, are areas of particular neglect in the present literature despite the prevalence of BDD in these groups (Phillips 2009, 2017). It would also be important to understand the role of wider diversity variables (e.g., sexuality and cultures). Neurodivergence was not explored here despite emerging recognition that it could be linked to body image difficulties, such as in the context of autism (Longhurst 2023). Given the conflicting evidence of whether autistic people can use mental imagery (Bled et al. 2024; King et al. 2024; Lau‐Zhu, Stacey, et al. 2024), BDD‐relevant imagery should be directly assessed in this population.

### 4.2 Limitations of This Review

There was marked heterogeneity between studies, especially in relation to BDD and imagery measurement, procedures for generating/utilising imagery, reporting of data and study quality. These inconsistencies precluded pooling trends and effect sizes, limiting our analysis to narrative methods of synthesis. The intervention studies alluded to mechanisms but were mostly focused on efficacy, so are unable to be conclusive about why and how imagery is related to BDD from these studies. The available evidence provides preliminary indication that demographic and contextual differences in imagery characteristics are subtle but important; however, this claim cannot be conclusive as no available studies have explored content or mechanistic differences in BDD imagery cross‐culturally. Additionally, findings were not weighted by study quality or design, which could be considered in future as more research accumulates. Our research team included specialists in imagery (DV and ALZ) and the originator of a theoretical model for BDD incorporating imagery (DV), which have informed the imagery focus. While this helped contextualise the findings within a cognitive‐behavioural perspective, other models could also be valuable in future reviews.

### 4.3 Conclusion

This systematic review highlights the important role of mental imagery in the maintenance and potential onset of BDD. Despite methodological heterogeneity across studies, this Meta‐Integration consistently characterised imagery in BDD as vivid, intrusive and distressing, primarily reflecting negative self‐perceptions and body appraisals. Specific content reflected the onset and/or course of one's perceived flaws, especially regarding their early experience of appearance‐related criticism and the familial and cultural backdrop within which these were experienced. Intrusive imagery also appears an accessible target for psychological interventions in BDD where innovations are much needed. An increased focus on imagery‐related processes in BDD has the potential for improved understanding of BDD, advancing early detection and intervention, particularly for individuals with trauma histories and/or in adolescence, where susceptibility to BDD appears greatest.

## Author Contributions

**Sean Hill:** conceptualisation, methodology, validation, investigation, resources, writing – original draft, writing – review and editing, visualisation and project administration. **Matthew Hotton:** conceptualisation, methodology, validation, writing – review and editing and supervision. **Martha Wallace:** validation, methodology, investigation, resources and writing – review and editing. **David Veale:** conceptualisation, methodology, validation, writing – review and editing, visualisation and supervision. **Alex Lau‐Zhu:** conceptualisation, methodology, validation, writing – review and editing and supervision.

## Funding

David Veale is partly funded by the National Institute for Health Research (NIHR) Oxford Health Biomedical Research Centre at South London and Maudsley NHS Foundation Trust and King's College London. Alex Lau‐Zhu is supported by a Medical Research Council Clinician Scientist Fellowship [MR/Y009460/1] and also reports funds from a John Fell Fund from the Oxford University Press [0014097], National Institute of Health Research (NIHR) Oxford Health Biomedical Research Centre, Oxfordshire Health Services Research Committee Research Grant and Committee for Children Junior Research Fellowship at Linacre College.

## Conflicts of Interest

The authors declare no conflicts of interest.

## Supporting information


**Data S1:** Qualitative synthesis deriving descriptive and analytical themes from coding labels assigned to verbatim results/findings of each included study, in accordance with Thomas and Harden (2008).

## Data Availability

The data that supports the findings of this study are available in the supplementary material of this article.

## Appendix A PROSPERO Protocol Amendments

The original PROSPERO protocol, published on 13 September 2023, detailed the following four questions as the ‘Outcomes to be Analysed’:

1. How has mental imagery of the self been assessed in BDD?
2. What are the characteristics of mental imagery in individuals with BDD and what are the hypothesised mechanisms?
3. How has mental imagery been utilised in interventions for BDD?
4. What is the quality of the evidence supporting the centrality of imagery in Veale et al. (1996)'s model of body dysmorphic disorder?

One deviation to the original protocol was made on 3 June 2024. During initial screening it was clear Items 1, 3 and 4 each asked for information already being gathered for Item 2, (‘What are the characteristics of mental imagery in individuals with BDD and what are the hypothesised mechanisms?’). In addition, Item 2 also denotes the central review question for this article. As such, Items 1, 3 and 4 were collapsed such that only Item 2 remained as the ‘outcome to be analysed’.

## Appendix B Full Search Strategy

### B.1 A.1. Concept #1. Body Dysmorphic Disorder (BDD)

BDD or ‘body dysmor*’ or ‘body dissatisfaction’ or ‘body satisfaction’

### B.2 Concept #2. Mental Imagery

‘mental imag*’ or images or imagery or imagination or visualisations or visualizations or aesthetic or aesthetic

### B.3 Main Search

Concept #1 and Concept #2

## Appendix C Author Background Position Statement

In systematic reviews using narrative methods of synthesis, interpretation plays a key role in shaping conclusions. Given the subjective nature of these methods, the authors' professional backgrounds and experiences may influence how the evidence is analysed. This statement provides transparency about our backgrounds to ensure readers can critically assess any potential biases in our interpretation and findings.

The lead author SH was involved in all stages of project design, data collection and analysis. At the time of writing, SH was a Trainee Clinical Psychologist who identified as White British, male, without lived experience of BDD. SH was employed by the National Health System (NHS) in the United Kingdom and had experience of providing psychological interventions to adults (age 18–65) and older adults (age 65+) with body image‐related concerns using a CBT model.

Author MH served as a supervisor for the review. He was a Clinical Psychologist who identified as a White British male, without lived experience of BDD. He has worked clinically with individuals with body image concerns, particularly relating to cleft or craniofacial conditions.

Author DV served as a supervisor for the review. He was a Consultant Psychiatrist who has worked with BDD clinically and in research. He authored a cognitive‐behavioural model of BDD including imagery.

Author MW served as an independent reviewer for the review. She was a Trainee Clinical Psychologist who identified as a White British female, without lived experience of BDD. She has worked delivering psychological care and interventions to individuals of all ages and across both physical and mental health settings.

Author ALZ served as lead supervisor for the review. He is a Clinical Psychologist and Researcher who identifies as a Peruvian‐Chinese male, uses pronouns he/they, without lived experience of BDD. He primarily works clinically with adolescents with trauma and anxiety‐related presentations in the UK NHS and conducts a clinical research program on understanding mental imagery's role in youth mental health.
