# Supplementary material for: Mental Imagery of the Self in Body Dysmorphic Disorder: A Mixed‐Methods Systematic Review
Source: Clin Psychol Psychother. 2026 Feb 15;33(1):e70229. doi: 10.1002/cpp.70229 (PMC12907616; doi:10.1002/cpp.70229)
Supplement: Supplementary file 1 — Data S1: Qualitative synthesis deriving descriptive and analytical themes from coding labels assigned to verbatim results/findings of each included study, in accordance with Thomas and Harden (2008). [file CPP-33-e70229-s001.docx]

| *Supplementary file.. Qualitative synthesis deriving descriptive and analytical themes from coding labels assigned to verbatim results/findings of each included study, in accordance with Thomas & Harden (2008).* | | | | |
| --- | --- | --- | --- | --- |
| **Quote** | **Article** | **Initial code** | **Descriptive theme** | **Analytical theme** |
| *pleasant sessions of exercise workout (past and present) focusing on muscles and feeling strong* | Ghaderi et al., (2022) | strong feeling, muscle body part, past and present time location | Body part of imagery | Content |
| *misfit of different outfits, being big, heavy and clumsy,* | Ghaderi et al., (2022) | weight and shape, outfits |  |  |
| *bad skin, pimples, big belly, aging body, being boney with many scars, being too fat,* | Ghaderi et al., (2022) | skin, blemishes, stomach, age, bones, scars, fat |  |  |
| *looking bigger than one actually is,* | Ghaderi et al., (2022) | imagery bigger than perceived self |  |  |
| *the aspect of the appearance that received the criticism went on to become an area of concern and distress for the participant.* | Craythorne et al., (2022) | congruence between area bullied for and bdd area of concern |  |  |
| *Participants’ responses were subsequently coded for the presence or absence of each factor (coded as 0 or 1).* | Onden-Lim & Grisham, (2012) | Coding was to presence and absence of certain features |  |  |
| *The most frequently reported body parts of concern were the stomach (33.8 %), skin (30.7 %), weight (29.2 %), thighs(24.6 %), legs (13.8 %), arms (13.8 %), hair (13.8 %), body proportion/shape (12.3 %), overall appearance of face(10.8 %), and breasts (10.8 %).* | Onden-Lim & Grisham, (2012) | stomach most common, skin and weight common, then thighs legs arms hair, body shape face and breasts |  |  |
| *4 (7.2 %) reported images that containedtheir body ideal* | Onden-Lim & Grisham, (2012) | Although uncommon, some imaged their ideal body |  |  |
| *(e.g., “me with larger chest, smaller thighsand stomach fat”), 3* | Onden-Lim & Grisham, (2012) | imagery of ideal chest, thighs and stomach |  |  |
| *Two referred to specific body sensations as well (e.g., my spots popping).* | Cooper & Osman, (2007) | some also mentioned body sensations such as spots popping |  |  |
| *For women, typical features of con-cern were their skin, eyes, hair, teeth, and geni-talia.* | Cooper & Osman, (2007) | women area of concern more skin eyes hair teeth and genitalia |  |  |
| *For the men, eyes, hair, and genitalia werealso common concerns, as well as their nose andhead size* | Cooper & Osman, (2007) | male areas of concern were eyes hair genitalia nose and head size |  |  |
| *Participants in the BDDgroup were also more conscious of their internalbody sensations* | Cooper & Osman, (2007) | bdd patients more conscious of internal body sensations than controls |  |  |
| *and involved facial and bodilyfeatures that took up a greater proportion of thewhole image* | Cooper & Osman, (2007) | compared to controls, bdd imagery involved a greater proportion of facial and body images in the total image area |  |  |
| *Often it is theparticipants’ favourite features, which are most identified with theself, that are then later viewed as defective:* | Silver & Reavey (2010) | often the “favourite features” of the childhood self are the current body areas of concern in bdd |  |  |
| *definitely different to my left eye, and look at myeyes. I started worrying less about my skin, and looking more atmy eyes.* | Silver & Reavey (2010) | when focusing on feeling one body area of concern is bad, eg their eyes being asymmetrical ,then look at other parts of their face and judge whether they are symmetrical. so the imagery then leads to more generalised negative self perceptions |  |  |
| *percentage occupied by the body/body parts,* | Onden-Lim & Grisham, (2012) | no correlation between bdd severity and focus on body / body parts |  |  |
| *‘Can he see my face? Is my face being ugly? . . . whatmy hair’s doing, erm . . ., “Why am I bending over likethis?” or my fat’s like bunching together (* | Stechler & Henton, (2022) | during intimacy, imagery of body areas of concern eg seeing own fat rolled up or trying to iamgine what hair and face looks like from partners perspective |  |  |
| *described seeing an array ofimages of the bodies of “beautiful women”* | Stechler & Henton, (2022) | one person described during intiamacy, seeing intrusive imagery of “beautiful women” like a slideshow in their mind, alongside the disembodied, cut off experience |  |  |
| *The median number of modalities reported for theimages/impressions was 2.00* | Cooper & Osman, (2007) | on average, imagery happened in 2 sensory modalities for bdd group, and 1 modality for control group | Modality of imagery |  |
| *This difference was sig-nificant U = 98.50, p = .04).* | Cooper & Osman, (2007) | bdd patients experience imagery in more modalities than non-bdd controls |  |  |
| *The visual modality was the most commonlyreported sensory modality for both patients andcontrols* | Cooper & Osman, (2007) | visual modality the most common for imagery |  |  |
| *followed by the organic modality* | Cooper & Osman, (2007) | internal/organic modality second most common imagery |  |  |
| *Thelatter consisted of feelings of anxiety, such astingling sensations in the body part of concern anda feeling of butterflies in the stomach* | Cooper & Osman, (2007) | organic/internal imagery was experienced as anxiety body sensations, like triggering the body areas of concern or butterflies in stomach feeling |  |  |
| *No parti-cipants reported gustatory or olfactory sensations.* | Cooper & Osman, (2007) | no gustatory or olfactory imagery reproted by anyone |  |  |
| *Visual 15 83%) 14 100%)* | Cooper & Osman, (2007) | every bdd early memory was in the visual modality |  |  |
| *Organic internal) 13 72%) 12 85%)* | Cooper & Osman, (2007) | 85% of bdd imagery was in the organic internal modality |  |  |
| *Auditory 1 6%) 8 57%)* | Cooper & Osman, (2007) | 57% of bdd imagery had the auditory modality |  |  |
| *Kinaesthetic 1 6%) 6 43%)* | Cooper & Osman, (2007) | 43% of bdd imagery involved the kinaesthetic/movement modality |  |  |
| *Cutaneous 1 6%) 5 36%)* | Cooper & Osman, (2007) | 36% of bdd imagery involved tactile / cutaneous modlaity |  |  |
| *Gustatory 0 0Olfactory 0 0* | Cooper & Osman, (2007) | no bdd imagery was in the gustatory or olfactory modality |  |  |
| *reported for the BDD group early memories was2.50* | Cooper & Osman, (2007) | on average, imagery was experienced in 2.5 modalities per time for bdd people |  |  |
| *There was also nocorrelation between dysmorphic concern and the number ofreported imagery modalities* | Onden-Lim & Grisham, (2012) | no correlation between number of modalities and bdd severity |  |  |
| *Since few participants reportedimagery modalities other than visual* | Onden-Lim & Grisham, (2012) | very few people report imagery in a mode other than visual |  |  |
| *and thatsame feeling it’s just always there ready to pounce.* | Silver & Reavey (2010) | person implies that more anxiety, more sensory modalities. it is only when they feel strong anxiwty about their distorted imaery that they start to feel it in their skin |  |  |
| *the described images by thefirst author and another independent researcher created animpression of a variety of scenarios* | Ghaderi et al., (2022) | participants reported different impressions and perspectives and things within the imagery | Perspective of imagery |  |
| *looking criticallyat one’s body in the mirror,* | Ghaderi et al., (2022) | mirror, body shape |  |  |
| *frequency of mental imagery in general (i.e., how oftenthey have various mental images in mind) was not related tobody dissatisfaction* | Ghaderi et al., (2022) | more often imagery not associated with more dissatisfaction |  |  |
| *However, we found a significant cor-relation between the frequency of body-related imagery andbody dissatisfaction* | Ghaderi et al., (2022) | but, body-focsued imagery associated with more dissatisfaction |  |  |
| *sizeable negative correlationbetween positive valence of body-related mental images andbody dissatisfaction.* | Ghaderi et al., (2022) | body dissatisfaction still get positive imagery too |  |  |
| *I just couldn’t cope with looking in the mirror and seeingthe left hand side of my face* | Craythorne et al., (2022) | found mirror gazing hard, to be the first noticable feature of bdd |  |  |
| *When a photo is taken of me I have to spend ages looking atit and it makes me feel disgusting and I feel disgusting nowthinking about different photos I’ve looked at with me in.I look deformed and it makes me worry because I must lookthat vile in real life* | Craythorne et al., (2022) | seeing photos of self seems to trigger imagery of self in real life in critical way |  |  |
| *16 (29.1 %) reported imagesthat contained another person/people* | Onden-Lim & Grisham, (2012) | 30% of imagery involved another person or people |  |  |
| *Images often contained more than one category (e.g., thecurrent self and another person/other people).* | Onden-Lim & Grisham, (2012) | images often involved self, others, and other elements rather than just one singular image |  |  |
| *The remain-ing images containing another person/other people involvedpositive (17.6 %) or neutral (5.9 %) descriptions, often wherethe other person/people was judged as very attractive.* | Onden-Lim & Grisham, (2012) | some imagery with tohers in was neutral, but most often involved the toher people being very attractive |  |  |
| *The majority of the sample (63.8 %) viewed their imagesfrom an overall observer perspective* | Onden-Lim & Grisham, (2012) | most imagery was from the observer perspective |  |  |
| *a smaller proportion of participants (27.7 %)viewed their images from an overall field perspective* | Onden-Lim & Grisham, (2012) | less, but still about a quarter, of imagery was from the overall field perspective |  |  |
| *and the remaining partic-ipants (8.5 %) viewed their images from both perspectivesequally.* | Onden-Lim & Grisham, (2012) | some people experiened imagery from the observer and the field perspective |  |  |
| *the majority of participants’ imagesappeared in the context of a social comparison/situation,* | Onden-Lim & Grisham, (2012) | the majority of imagery was in the context of, in real life, experiencing a social comparison |  |  |
| *Focus of Attention in the Image. All patients reported paying most attention to their appearance in the image* | Cooper & Osman, (2007) | all 18 said that imagery focused on their appearance |  |  |
| *Eight patients "always" thought that their image/impression was a fact,* | Cooper & Osman, (2007) | 8/18 people felt their imagery/thoughts were an absolute fact |  |  |
| *the remainder of the patients (10) reported that they could “sometimes” see it either as a distortion or “at a distance from what was really happening.”* | Cooper & Osman, (2007) | 10/18 sometimes saw it as a distortion and sometimes as fact, or had some other level of insight into not fully believing the imagery as fact |  |  |
| *and external appearance* | Cooper & Osman, (2007) | bdd patients more conscious of their external appearance than controls |  |  |
| *The observer/mirror perspectiveThe median ratings for perspective of the images/impressions were 3.00 viewing myself completelyfrom an external viewpoint, as if through the eyesof another, IQR = 3.00 to 3.00* | Cooper & Osman, (2007) | most bdd people said the image was themselves from a third person perspective, wheras control group said it was themselves from their own eyes |  |  |
| *viewing myself completely from myown eyes, as if in a mirror, IQR = 73.00 to 73.00)for the control group. It is* | Cooper & Osman, (2007) | so bdd more likely to see an external picture of themselves, controls more likely to see just their usual visual of self |  |  |
| *All BDDparticipants saw themselves from an observerperspective, while all control participants reportedthe mirror perspective.* | Cooper & Osman, (2007) | difference between bdd and controls bdd all reported seeing themselves in third person, all controls saw themselves from a mirror perspective |  |  |
| *These memories werealso mainly viewed from an observer perspective* | Cooper & Osman, (2007) | intrusive memories in bdd mostly observed from a third person perspective |  |  |
| *sensory similarity reported between thespontaneous image/impressions and the asso-ciated memories was 60%,* | Cooper & Osman, (2007) | 60% similarity between the imagery and associated memories |  |  |
| *if we are having sex [. . .] I see pictures in my head oflike other girls and like just sort of, just sort of start* | Stechler & Henton, (2022) | during sex, person sees images of other girls and that makes them feel gross about themself |  |  |
| *like a slideshow of bodies, beautiful women thatI don’t look like.* | Stechler & Henton, (2022) | during intimacy, person describes slideshow of other girls they don’t look like and feel disgusting and bad |  |  |
| *In participants’ narratives, BDD seemed to take onan identity of its own—as though there were three ina relationship,* | Stechler & Henton, (2022) | during intimacy, bdd acts like a third person in the room, one who is more superior and sabotaging and wants to control or restrict freedom |  |  |
| *certain ways. BDD was a bully or abuser—one participant suggested that like a bird it “pecksat you” in a way that sounded as if it might feel likea physical pain* | Stechler & Henton, (2022) | bdd imagery can feel like the abuser itself, as it judges, has been referred to as like physical pain, and feels like a constant set of “sharp jabs” |  |  |
| *joke. It seemed there might bea link between an inadequate sense of self and inade-quate parts of their body, which they described in termssuch as “awful,” (Mia) “giant” (Samantha, Grace) or “rub-bish”* | Stechler & Henton, (2022) | link between adequacy as a person and adequacy of the parts of body they dislike, ie they are inadequate and therefore they have these flaws |  |  |
| *The doubt she dwelled upon regarding whethershe has BDD appears to be due to the inseparability of herperceived self and objective self. In other words, her perceiveddistortions are irrefutable, in that she does not consider themto be distortions.* | Craythorne et al., (2022) | indiscrimination between perceived and objective self barrier to insight |  |  |
| *One person noted that it triggered flashbacks, another that it was an accurate representation.* | Cooper & Osman, (2007) | imagery triggered flashbacks in one person out of 18 |  |  |
| *Participants described disgust and a sense of detach-ment or disembodiment during physically intimatemoments with their partners.* | Stechler & Henton, (2022) | or trigger, partner described that during moments of intimacy, they experienced detachment or disembodiment from themselves |  |  |
| *adopting a third-person perspective* | Stechler & Henton, (2022) | every woman interviewed said they felt they saw themselves from a third person perspective when intimate with a partner |  |  |
| *Their preoccupied thoughts and worries ran-ged over their bodies as a visual stimulus* | Stechler & Henton, (2022) | their preoccupation with thoughts and images meant they were less of an active participant in the act of intimacy itself |  |  |
| *The use of the photographs was a powerful way of capturingfeelings that could not always be articulated* | Silver & Reavey (2010) | using old photographs seems to evoke imagery about emotion and content, and bring out feelings in a way that people find hard to articulate without guidance |  |  |
| *From this initial night-mare of a traumatic skin eruption,* | Silver & Reavey (2010) | from one experience of bad imagery about body area of concern, this perception gets distorted over time to more exaggerated and negatively perceived defects |  |  |
| *All the participants reported at least one body-related men-tal image* | Ghaderi et al., (2022) | All participants reported at least one experience of body-related imagery | Presence of imagery |  |
| *Fifty-five (84.6 %) participants reported experiencing recur-rent intrusive imagery.* | Onden-Lim & Grisham, (2012) | most people, 84.6%, experienced intrusive recurrent imagery |  |  |
| *All of the BDD patients reported experiencingeither spontaneous images n = 17, 94%) or othersensory impressions n = 1, 6%) when worried oranxious about their appearance.* | Cooper & Osman, (2007) | all bdd patients experienced spontaneous imagery when worried about appearance |  |  |
| *Of the controlparticipants, 15 83%) reported either sponta-neous images n = 6, 33%) or sensory impressionsn = 9, 50%).* | Cooper & Osman, (2007) | in general population/non-bdd controls, 83% experienced spontaneous intrusive imagery or sensory experiences too |  |  |
| *There was no difference between thepatients and controls in reported frequency ofspontaneous images/impressions* | Cooper & Osman, (2007) | do significant difference between frequency of imagery, between the bdd patients and the controls |  |  |
| *Of those experiencing spontaneous images/impressions, 17 94%) in the patient group and 746%) in the control group reported these to berecurrent.* | Cooper & Osman, (2007) | twice as many bdd patients compared to controls said the imagery was recurrent |  |  |
| *there was nosignificant correlation found between dysmorphic concern(indexed by BICI) and image frequency* | Onden-Lim & Grisham, (2012) | no correlation between bdd concern and frequency of images |  |  |
| *and to put on favorite jeans and feeling how well theyfit.* | Ghaderi et al., (2022) | ideal of a future with better body | Time location of imagery |  |
| *future nice haircuts and hairstyles* | Ghaderi et al., (2022) | future |  |  |
| *being on the move (e.g., onthe beach)* | Ghaderi et al., (2022) | movement, outside objects |  |  |
| *She experiences a feelingof disgust if she does inspect a photograph of herself, and thisdisgust is also evoked by memories of her appearance inphotographs too, suggesting she may be haunted by residualdistortions.* | Craythorne et al., (2022) | photos can be reminders of appearance related memories and bring this back |  |  |
| *factors included the subjectdepicted in the images and temporal quality (i.e., past self,present self, future self, ideal self, other).* | Onden-Lim & Grisham, (2012) | authors found theme of temporal quality of images, and content of images |  |  |
| *Thirty-four participants (61.8 %) reported images that containedtheir present self* | Onden-Lim & Grisham, (2012) | most imagery was in the present version of self |  |  |
| *(5.5 %) reported images that containedtheir self in the past (e.g., “I see my body when I was inmiddle school”),* | Onden-Lim & Grisham, (2012) | small percentage of imagery in the past eg at school |  |  |
| *2 (3.6 %) reported images of them-selves in the future (e.g., “myself—older and bigger”).* | Onden-Lim & Grisham, (2012) | some in the future but this even rarer than the past |  |  |
| *Images tended to be viewed as moving films(46.7 %) or still photos (42.2 %),* | Onden-Lim & Grisham, (2012) | imagery was moving films or still photos |  |  |
| *with the remaining viewedas a series of different photos or snapshots (11.1 %).* | Onden-Lim & Grisham, (2012) | 11% ish were a series of different photos and snapshots |  |  |
| *Inspection of the data suggested thatthere was generally a close match betweenreported modalities, i.e., patients who experi-enced a particular modality in their images/impressions were also likely to experience thatmodality in the associated memory.* | Cooper & Osman, (2007) | close match between the modalities remembered in the early memory, and those experienced in imagery. ie if it was a visual memory, the visuals are seen in imagery |  |  |
| *He’s a good-looking chap aint he?* | Silver & Reavey (2010) | using photograph imagery, people used third person accounts to describe their past self, eg “he is a good looking chap” or “i don’t know who that girl is” instead of using the terms “i” and “me” |  |  |
| *future weightloss* | Ghaderi et al., (2022) | future |  |  |
| *past experiences of pregnancy,* | Ghaderi et al., (2022) | past experience, pregnancy |  |  |
| *The BDD group reported images/impressions thatwere visually more vivid* | Cooper & Osman, (2007) | bdd imagery was significantly more vivid than for the controls | Vividness of imagery |  |
| *more detailed* | Cooper & Osman, (2007) | bdd imagery also had higher detail and visual acuity/sharpness |  |  |
| *The BDD images/impressions alsoinvolved organic sensations that were more vividU = 6.00, p = .002) and intense* | Cooper & Osman, (2007) | bdd imagery for organic/internal feeling was also more vivid and intense |  |  |
| *No correlation was foundbetween dysmorphic concern and vividness, perspective ordetail (colour, brightness, extent of 3D representation(depth),* | Onden-Lim & Grisham, (2012) | no correlation between bdd severity, and vividness perspective or detail about images |  |  |
| *Which despite signifi-cant variations were readily divided into a positive or a nega-tive theme.* | Ghaderi et al., (2022) | Imagery is positive or negative | Negative emotions | Consequences and maintenance patterns |
| *being mindful of the body (e.g., whilewalking and sensing the wind),* | Ghaderi et al., (2022) | mindful |  |  |
| *sensing freedom and strength* | Ghaderi et al., (2022) | freedom and strength |  |  |
| *current negative consequence of past pregnancies on thebody,* | Ghaderi et al., (2022) | negative past present today |  |  |
| *and a dysfunctional body.* | Ghaderi et al., (2022) | negative imagery on body function |  |  |
| *the occurrence of both negative andpositive mental images* | Ghaderi et al., (2022) | imagery can be positive and negative |  |  |
| *between negative valenceof images and body dissatisfaction* | Ghaderi et al., (2022) | more body dissatisfaction, more likely to see imagery as negative |  |  |
| *targeting negativebody-related mental images* | Ghaderi et al., (2022) | should focus on the negative images as these are associated with body dissatisfaction more than imagery in general |  |  |
| *Most images containing another person/other people containednegative descriptions (76.5 %);* | Onden-Lim & Grisham, (2012) | imagery involving others was mostly negative |  |  |
| *they involved themes includinglooks of disgust form another person/other people, and/or otherpeople talking about or laughing at the individual.* | Onden-Lim & Grisham, (2012) | imagery involving others was mostly about the other person/people critiquing and comparing them |  |  |
| *Participants also rated the majority of images(82.5 %) as negative in emotional tone* | Onden-Lim & Grisham, (2012) | most imagery was negative in emotional tone |  |  |
| *with some images (14.0 %) ratedas positive in emotional tone (i.e. rated their images from +1to +3), and the smallest proportion of images rated as neutral(3.5 %).* | Onden-Lim & Grisham, (2012) | about 14% of images were positive |  |  |
| *All reported disadvantages. Thirteen reported that the image made them feel more self-conscious, eight reported that the image made them feel emotionally distressed or depressed, while six reported that it decreased their self-confidence and/or self-esteem.* | Cooper & Osman, (2007) | all 18 said that it had disadvantages, stating imagery made them more self conscious, emotionally distressed, depressed, decreased self-confidence and esteem |  |  |
| *Six reported that it affected their behavior (including ability to go out or get on with life).* | Cooper & Osman, (2007) | one third of participants said imagery affected their behaviour broadly, such as inability to get out on get on with life |  |  |
| *One patient noticed that attending to the image distorted it more, another that it increased his or her focus on appearance, and two that it reinforced the negative way they felt about themselves.* | Cooper & Osman, (2007) | attention paid to imagery increased the distortion more, increased focus on appearance, and reinforces negative self-perceptions |  |  |
| *five used negative comments and reactions from other people, three used body sensations, three used their own thoughts, two used their appearance, and one used the media.* | Cooper & Osman, (2007) | negative judgments also maintained by seeing negative reactions from others, body sensations, own thoughts, and the media |  |  |
| *Four patients reported advantages to their negative thoughts. Three said that it motivated them to change their appearance and one that it prevented him or her from becoming arrogant. All reported disadvantages* | Cooper & Osman, (2007) | similar opinion of whether negative thoguhts have advantages/disadvantages experiences as with imagery |  |  |
| *Thirteen patients “always” thought that their thought was a fact, while the remainder of the patients (five) reported that they could “sometimes” see it either as a distortion or “at a distance from what was really happening”.* | Cooper & Osman, (2007) | more people thought negative thoughtd were accurate/factual than thought imagery was accurate/factual (8/18 for imagery, 13/18 for thoughts) |  |  |
| *Being seeninvolved at times a sense of unbearable, excruciatingexposure.* | Stechler & Henton, (2022) | being seen naked/semi-naked by another seen as unbearable and excrutiating exposure |  |  |
| *In parallel, thisdistress seemed to manifest in the way participantsrecalled their experiences during the interviews* | Stechler & Henton, (2022) | describing imagery content in interview brought up intense emotions in the participants |  |  |
| *The participant’s concern was that in the showerher partner could “really look” at and see the “real”“it.”* | Stechler & Henton, (2022) | participants described worrying that if partners saw their naked body they would see “the real” them |  |  |
| *feel as if my flawswere deformities* | Craythorne et al., (2022) | escalation from appearance flaws to deformities |  |  |
| *It made me feel kindof good that other people had it and I started to read about itmore and it would always make me feel* | Craythorne et al., (2022) | validating but also saddening |  |  |
| *My BDD flares up considerably whenever I am stressed oranxious but even when I am not, it is there with me constantlyevery day… you asked how often I thought about my bodyimage and I can honestly say that it would be impossible to saybecause it feels like the thoughts are there almost all the time.It would be easier to let you know how often I don't thinkabout it. It is quite often my waking thought and I dreamabout it regularly.* | Craythorne et al., (2022) | bdd related symptoms and imagery worsen when more stressed and anxious |  |  |
| *The majority of images were rated by the participants asnegative in emotional tone* | Onden-Lim & Grisham, (2012) | the majority of imagery content was negative in emotion |  |  |
| *except for images containing idealselves, of which the majority were described as having apositive emotional tone.* | Onden-Lim & Grisham, (2012) | the majority of imagery about ideal body shape was described as positive imagery |  |  |
| *The remaining images containing the present self(22.6 %) contained neutral content (e.g., “I see myself without ashirt”).* | Onden-Lim & Grisham, (2012) | some imagery was neutral in content ie not better or worse |  |  |
| *On the other hand, those with images containing a bodyideal (100 %) or the self in the past (66.0 %) described the selfin desirable or neutral terms* | Onden-Lim & Grisham, (2012) | imagery of the past, and of the body ideal, were perceived in good or neutral terms |  |  |
| *All patients reported either a spontaneous image (n = 17) or a sensory impression (n = 1) when worried or anxious about their appearance.* | Cooper & Osman, (2007) | all participants experienced intrusive imagery when worried or anxious about appearance |  |  |
| *All patients also reported one or more negative thoughts when worried or anxious about their appearance.* | Cooper & Osman, (2007) | as well as imagery, all participants experienced negative thoughts when anxious/worried about appearance |  |  |
| *Twelve of the patients had negative thoughts about the image.* | Cooper & Osman, (2007) | 2 thirds of people had negative thoughts about their imagery |  |  |
| *Ten patients reported paying most attention to their appearance in the thought.* | Cooper & Osman, (2007) | negative thoughts also increased attention to self, and to imagery of the self |  |  |
| *Four reported being aware of the “whole thought,” while two reported being aware of how they looked in the mirror. One reported most awareness of tension pain.* | Cooper & Osman, (2007) | negative thoughts increased some imagery on pain feelings eg tension |  |  |
| *According to questionnaire scores the BDDpatients were more depressed BDI-II: U = 8.00,p < .001), had lower self-esteem RSE: U = 4.50, p< .001), and had higher fear of negative evaluationby others FNE: U = 24.00, p < .001) than thecontrol participants* | Cooper & Osman, (2007) | bdd patients have more depression, low self esteem and more negative evaluation fears than controls |  |  |
| *BDD patients' ratings were significantlymore negative U = 28.50, p < .001) than thecontrol groups' ratings.* | Cooper & Osman, (2007) | imagery has significantly more negative emotional tone for bdd people than controls |  |  |
| *Even though I tried to hide itand act unbothered by them* | Craythorne et al., (2022) | person acted unbothered by trauma at the time | Safety-Seeking Behaviours |  |
| *But I think it wasquite soon after that I tried to dig a mole on my right forearmout with a sharp stone in the playground… I also tried to diga mole out of the rear of my thigh, but that was not successful.* | Craythorne et al., (2022) | self surgery as child to correct the perceived defect |  |  |
| *after the phone call I was staring in the mirror* | Craythorne et al., (2022) | checking behaviour immediately after the trauma event |  |  |
| *The longer I looked [in the mirror] the less it made sense whatI was looking at* | Craythorne et al., (2022) | mirror gazing more distorts perception more |  |  |
| *the features seemed to be much moreindividual rather than looking at a face as a whole…* | Craythorne et al., (2022) | mirror gazing increases focus on specific body areas |  |  |
| *whether image suppression predicted dysmorphic concernafter controlling for depression, anxiety, and stress.* | Onden-Lim & Grisham, (2012) | imagery suppression significantly associated with bdd, even when controlling for depression, anxiety and stress |  |  |
| *Eleven reported wishing it would go away, that they were fed up with it, or that they told it to go away.* | Cooper & Osman, (2007) | 11/18 people wished the imagery would go away |  |  |
| *Eleven patients engaged in distraction (cognitive, behavioral, or both) to cope with the negative thoughts they experienced* | Cooper & Osman, (2007) | 11/18 people used distraction to try and cope with the imagery |  |  |
| *Two went to sleep, three engaged in checking, two avoided situations or activities, three engaged in self-talk (e.g., it isn’t true), one covered himself or herself up, and one waited for the thoughts to go away.* | Cooper & Osman, (2007) | strategies to distract from imagery included sleeping, checking, avoidance, self-talk, covering self up, waiting for thoughts to go away |  |  |
| *Three either had no coping strategies or simply waited for the image/impression to go away.* | Cooper & Osman, (2007) | 3 out of 18 had no coping strategies to manage the imagery/thoughts |  |  |
| *Eight wanted to feel better (less anxious, less depressed), six wanted to stop the image or thought from happening, two wanted to check that their looks hadn’t changed, two wanted to look better, two wanted to feel reassured, and one wanted to feel less self-conscious.* | Cooper & Osman, (2007) | reasons for coping strategies for imagery included wanting to feel better, stop the imagery, check that nothing had changed, feel reassured, and feel less self conscious |  |  |
| *Three patients reported advantages to this. One patient reported that it motivated them to change their appearance and two that it prevented them from becoming arrogant.* | Cooper & Osman, (2007) | 3/18 said imagery had benefits, because it either motivated them to change appearance or prevented them from becoming arrogant |  |  |
| *Role of Memory to Deal With Image. Seven patients used their memory to try to deal with the situation in their image/impression. Most thought of past positive experiences that they had had, while one person tried to think of people who were worse off than himself or herself.* | Cooper & Osman, (2007) | participants used memories to deal with the image in their head, eg think of past experuiences or think of people worse off than themselves |  |  |
| *Twelve of the patients had negative thoughts about the thought. Five reported wishing it would go away, that they were fed up with it, or that they told it to go away.* | Cooper & Osman, (2007) | negative thoughts were coped with in a similar manner to imagery, although there was more challenging/rationalising/bargaining with thoughts than for imagery |  |  |
| *.I was twisting my face, it was uncomfortable, then it was likethat actually sort of twisted it in my mind* | Silver & Reavey (2010) | one person describes almost an active process of distorting their image in their mind to make it worse as it made them feel better to see themselves as distorted as it represented their negative self-perceptions |  |  |
| *I can feel the lumps, never, yeh,never ever forgotten that moment, and that anxiety,* | Silver & Reavey (2010) | and then as the imagery gets more distorted, that increases their anxiety as the image is so strongly related to their self perception |  |  |
| *look young for your age, but your eyes are, you can tell your eyesaren’t like proper 19 year olds.’ And then I started looking at myeyes, but, I started looking at, more so, I started staring at myeyes, but I st* | Silver & Reavey (2010) | memory of people saying appearance related thing about the face, led to more mirror gazing focusing on that part of the face, which then led to more imagery and distorted perception as focused on it more |  |  |
| *shielding them also from the unpleasantreality of their perceived flaws.* | Stechler & Henton, (2022) | camouflaging body from partner also felt like it had the effect of protecting themselves and their partner from their perceived flaws |  |  |
| *Without make-up, there seemed to be a furthersense of shame or embarrassment, and hands becamelike the wardrobe door:* | Stechler & Henton, (2022) | implied that if not doing camouflaging behaviorus like hiding and make-up, the increased shame and embaressment from feeling exposed evoked more vivid and powerful imagery |  |  |
| *some participants were tearful, others seemed tohold a tense or hunched posture* | Stechler & Henton, (2022) | some people hunched over, cired, looked down, asked for a break, when recalling the imagery they experience whe nintimate with their partner |  |  |
| *All participants attempted to control and manoeuvretheir partner’s gaze as a means of managing their* | Stechler & Henton, (2022) | peoples safety seeking strategies as a result of feeling partner sees them, is to try and control and maneuveure their parterns gaze, eg by positioning themselves or using angles to try and manipulate what their partner could see |  |  |
| *(withlaughter, repetition, pauses, turning away) was feltto be indicative of the importance of these man-oeuvres for them,* | Stechler & Henton, (2022) | during interview recalling intimate times with partners, participants noted to do the same controlling maneuvuers in interview eg laughter, pausing, turning away, which was felt to be indicative of how emotion evoked by recalling the experiences |  |  |
| *; rather it was a numbexperience, disengaged from their bodily senses.* | Stechler & Henton, (2022) | during sex, bbd people said felt numbs and disengaged from sense, wanting it to be over |  |  |
| *We found a significant positive association between dysmor-phic concern and image suppression* | Onden-Lim & Grisham, (2012) | bdd concern correlated strongly with attempts to suppress imagery |  |  |
| *In contrast, another participant suggested that thelongevity of her relationship and the fact she hadhabituated her partner to all her “flaws” made the* | Stechler & Henton, (2022) | in contrast, someone said they felt partner seeing them felt more comfortable than a stranger because they felt partner had habituated to their flaws | Impact on relationships |  |
| *worrying in some way what I appear like or what I’mdoing or what he’s seeing or what he’s looking at* | Stechler & Henton, (2022) | the detachment leading to them less present in the intimacy, led people to worrying that they weren’t good at intimacy or that they were being judged by their partner for not being good at intimacy |  |  |
| *Making negative comparisonsbetween her own body and the moving sequence ofimages of other girls in her mind was so upsettingthat she was frequently unable to continue* | Stechler & Henton, (2022) | one person described imagery as so upsetting they had to stop the act of intimacy |  |  |
| *Sex was described as a mundane obligation* | Stechler & Henton, (2022) | sex felt like a mundane obligation due to the negative experiences related to bdd it evoked |  |  |
| *I didn’t really knowwether [sic] I actually had it or wether [sic] I was being ahypochondriac but I must have it or I’m really hideous and I’musing it as an excuse for my horrible face* | Craythorne et al., (2022) | confusion in bdd symptoms, not knowing if have bdd or if perceptions are true. sounds insight related | Self-worth |  |
| *This fragmentation is a great source of frustration andsadness for some participants.* | Silver & Reavey (2010) | bdd people therefore seem to have a fragmentation between their past and former self, with idealisations about the past, that add to present day sadness and frustrations about their image and self |  |  |
| *BDD patients were happywith their appearance, when participants were asked how theywould ideally like to look, their response indicated that theywanted to look like their actual self without the ‘defects.’* | Silver & Reavey (2010) | in general, bdd peoples ideal self is just their actual self but without their perceived defects. ie not a completely different self. places centrality on the perceived defect as the cause of all their problems |  |  |
| *cative of BDD per se, the unique factor seemed to bethe level of distress that accompanied these experi-ences,* | Stechler & Henton, (2022) | appeared that although feeling body conscious is a normal concept, it is the level of distress that accompanied this that seemed unique in those with bdd |  |  |
| *and the way these experiences formed part ofa wider set of distressing phenomena.* | Stechler & Henton, (2022) | and also, the way experience of body consciousness evoked a complex set of other distressing phenomena associated with self judgment, self concept, shame, etc |  |  |
| *inthe process of preparing themselves to go out or where theyanticipated social contact* | Onden-Lim & Grisham, (2012) | imagery occurred when people were preparing themselves to go out or before seeing others | Cultural and Societal Influences | Causes |
| *many participants statedthat a perfect image would mean improved self worth.* | Silver & Reavey (2010) | many people referred to an idea that a perfect body image represents perfect self worth. therefore in bdd seems a tight link between image and self worth |  |  |
| *Peter, who attempts to modify his appearance by morphingpictures of himself on digital cameras comments that:* | Silver & Reavey (2010) | due to body image representing self worth, for some people it seems actively distorting their self image reflects their negative view of their self |  |  |
| *My mum’s side of the family, they tend to be, they are quite vain* | Silver & Reavey (2010) | one bdd person described their family as very vain people, leading to them feeling they had a lot to live up to with regards to appearance |  |  |
| *construction of the self. Louise feels that within the Indian culturewomen are very much judged on their looks and are expected to bebeautiful aesthetic objects to be gazed at both by other men and byother women:* | Silver & Reavey (2010) | indian culture, women expected to be aesthetic objects so self image is important. therefore culture seemed important on how bdd develops |  |  |
| *Around half the participants suggested there wasa difference between the gaze of a stranger andtheir partner’s gaze.* | Stechler & Henton, (2022) | people felt their partners gaze was different to that of a stranger, and more connected to their emotional perceptions of self as well as physical | Judgment and Criticism |  |
| *‘Oh, he’s . . . ‘ I’m more critical of myself (Victoria)* | Stechler & Henton, (2022) | participants described feeling their partner were more critical of their body than a stranger would be, because the person is more critical of themselves than a stranger would be |  |  |
| *uncomfortable and exposing—embodying an objecti-fied sense of self as “deformed* | Stechler & Henton, (2022) | being intimate with partner felt exposing, which triggered feeling objectified, which triggered feeling deformed/fat/gross, and then getting self conscious during intimacy |  |  |
| *participants again seemed to dis-connect from identifying their body as their own,* | Stechler & Henton, (2022) | in intimate moments, participants described not seeing their body as their own, feeling detached from themselves |  |  |
| *by a cognitive focus on theappearance of their body and highly negative apprai-sals of its appearance.* | Stechler & Henton, (2022) | as well as feeling detached from their own body, also felt highly conscious of body appearance and highly negatively critical of it |  |  |
| *“I’m just outside watching”:* | Stechler & Henton, (2022) | one person described “watching from the outside” during intimacy, like it triggers an out of body experience due to the associated feeling of detachment |  |  |
| *Detaching from their bodies seemed to shield themand help them escape from a potentially threateningsituation:* | Stechler & Henton, (2022) | seemed detaching from their body was a way to shield themselves from the threatening situation they perceived from threat of judgment |  |  |
| *this participant was also led to question herself,* | Stechler & Henton, (2022) | experienced judgmental imagery leads person to question themselves and their abilities |  |  |
| *How is he finding . . . that bit of my body sexy?’ Likewhen he touches my bum, I’m like, ‘Seriously, it’sgiant [. . .] “* | Stechler & Henton, (2022) | mental mind reading during se “what is he thinking, what can he see, does he like this” |  |  |
| *The amount of criticisms she received may havecontributed to her feeling overwhelmed by the negativeperceptions other people had ascribed to her body* | Craythorne et al., (2022) | lots of criticism makes it overwhelming |  |  |
| *you have lots of beauty spots’ in a pleasantway referring to facial moles I had/have… [the boy], who alsohad facial moles, said something like ‘they’re not beauty spots,they’re moles’ in a very harsh and disparaging way… I don’trecall openly reacting in any specific way.* | Craythorne et al., (2022) | at time, took in content and judged the negative over the positive content as true |  |  |
| *I was so much like my auntie [name redacted]'who was viewed very negatively by the family* | Craythorne et al., (2022) | overt social comparison to others viewed negatively |  |  |
| *By describing her face as “horrible,” sheattaches her distorted perception to her objective appearance,reinforcing their inseparability* | Craythorne et al., (2022) | distorted perceptions maintained in description of self as horrible |  |  |
| *articipants with images containingthe self in the present (77.4 %) and future (100 %) typicallydescribed the self in undesirable terms* | Onden-Lim & Grisham, (2012) | present and future imagery was usually undesirable, eg being fat or frustrated |  |  |
| *in having verbal thoughtsabout weight or general appearance.* | Onden-Lim & Grisham, (2012) | when ruminating on verbal thoughts about weight and appearance |  |  |
| *Two wanted to distract themselves, one wanted to get away from negative judgments, and one wanted to achieve his or her potential in life.* | Cooper & Osman, (2007) | also some wanted to distract self or get away from negative judgments, by trying to cope with the imagery |  |  |
| *All patients formed judgments about themselves as a result of having the image/impression.* | Cooper & Osman, (2007) | all participants had a negative judgment about themselves due to having the imagery |  |  |
| *Eleven thought that they were unattractive, six that they were ugly, and five that they were inferior or worthless, and three described themselves as a freak.* | Cooper & Osman, (2007) | due to having imagery, participants felt they were unattractive, ugly, inferior, worthless or a freak |  |  |
| *Eleven patients looked in a mirror to help them form their negative self-judgments* | Cooper & Osman, (2007) | most people used mirror gazing to further evidence the negative judgments that the imagery generated in them |  |  |
| *unable to come to terms with the idea that they were no longer thechild they were* | Silver & Reavey (2010) | notion that in bdd, people unable to come to terms with idea they are not the child they once were |  |  |
| *free of not only blemishes and impurities, but freefrom the responsibilities of their adult self.* | Silver & Reavey (2010) | compared to now, bdd people may find it hard that they used to be blemish/impurity free and free of responsibility |  |  |
| *to currentperceptions of ugliness or defects may overlook the link betweenpast and current versions of selfhood* | Silver & Reavey (2010) | emotional tone negative due to comparison between perceived self today and idealisation of the past self which is perceived more attractive version of themself |  |  |
| *referring to a ‘real’ reflectionof their lost youth, but a presently constructed idealised fiction ofthe past.* | Silver & Reavey (2010) | comparison between current and former self more of an idealised fiction of the past rather than a real reflection |  |  |
| *it is argued that references to an idealised pastself are a way of explaining and justifying their dissatisfactionrelated to their present image.* | Silver & Reavey (2010) | the idealised past self also explains and justifies dissatisfaction with present self image |  |  |
| *wherethey were either not concerned about or were proud of their looks.* | Silver & Reavey (2010) | also relate idea that felt happier as children because were not preoccupied with their looks |  |  |
| *There is a radical discontinuity of the self over time. Theyounger, attractive self is constructed as being completely dissim-ilar from the older* | Silver & Reavey (2010) | “radical discontinuity” - young self perceived as good and attractive, current self old and ugly and referenced in the third person |  |  |
| *as they were envious of the younger self* | Silver & Reavey (2010) | envy towards younger self often expressed |  |  |
| *The narrative starts by Susan recollecting that shewas an archetypal teenager with bad skin* | Silver & Reavey (2010) | negative perceptions of former self often expressed in a stereotypical way, eg one person describing an archetypal spotty teenager when describing their former appearance flaws |  |  |
| *kids in the family, how good looking they all are, and how we* | Silver & Reavey (2010) | person remembers at family events, other family would ask how the children are doing and how good looking they are, therefore internalised appearance as an important part of self worth |  |  |
| *suggesting a feeling of shame in connection totheir bodies* | Stechler & Henton, (2022) | participants had a lot of shame around their bodies |  |  |
| *Throughtheir partner’s viewing their bodies were objectified(“it”) in the spatial and visual field.* | Stechler & Henton, (2022) | seeing their partners view their bodies felt like an objectification of them self and triggered them to view themselves from the third person |  |  |
| *Participants’ ownjudgement of their bodies seemed to find itself withintheir partners’ imagined viewpoint or appraisal;* | Stechler & Henton, (2022) | their imagery about their partners perceived appraisal from a third person view seemed to also capture the persons own negative views about themselves, ie they assume partners also see the flaws they see in self |  |  |
| *All but one participant spoke about the ways theyprevented themselves from being seen naked orundressing by their partner, in order to protect (“shel-ter” Samantha) themselves from the judgement orshame they might experience under the observationof their partner.* | Stechler & Henton, (2022) | safety seeking behaviours around hiding and camouflage reflect wanting to mask judgment and shame they feel about themselves |  |  |
| *.] I don’t want him to see it* | Stechler & Henton, (2022) | people referred to their bodies as external to themselves, eg words like “i don’t want him to see it (their body)”, rather than “i don’t want them to see me/my body” |  |  |
| *Some participants referred to sex as “performance”(Grace, Mia) influenced by internalized messagesabout how sex should be with a pressure to have anorgasm the marker of successful sex.* | Stechler & Henton, (2022) | cultural aspect of sex feeling like a performance to please the other person, contributed to why it felt so judgmental and so much criticism |  |  |
| *what if he saw me for who I, how I really was, like . . .what if one day he just woke up and seeing all theflaws that I saw (Lucy)* | Stechler & Henton, (2022) | imagery around sexual intimacy led to worrying about the relationship “what if he woke up one day and saw my flaws and left me” |  |  |
| *which caused great distress to a number of participants inthe study* | Craythorne et al., (2022) | bullying and distress historically is still distressing in present | Traumatic Memories and Early Experiences |  |
| *The criticisms were often made bypeers, friends and family members,* | Craythorne et al., (2022) | criticism from peers, friends and family |  |  |
| *Criticismswere often extensive and were not limited to one aspect of theparticipants’ appearance.* | Craythorne et al., (2022) | bullying was not just appearance related |  |  |
| *. I was literally verbally “attacked” from head totoe;* | Craythorne et al., (2022) | bullying about appearance felt like being attacked |  |  |
| *my hair, my nose, my moles, my body hair, my weightand size, my “lack of curves”…* | Craythorne et al., (2022) | can quote and list bullying criticism in detail |  |  |
| *these comments cut me to thecore and I stored them deep within the corners of my brain,* | Craythorne et al., (2022) | bullying was remembered and very painful |  |  |
| *ruminating on them even to this day* | Craythorne et al., (2022) | bullying from past still ruminated on today |  |  |
| *By usingevocative words such as “insults,” “ridicule” and “attacked,”Angelina helps us to realise how adverse these incidents ofbullying were for her.* | Craythorne et al., (2022) | use of attacking language shows the harm caused |  |  |
| *Well I’ve-I’ve-I’ve been bullied since I was in year three upuntil year eleven [aged approximately 7-16 years]…* | Craythorne et al., (2022) | bullying was for many years, nearly 10 years |  |  |
| *… I had peopleanonymously messaging me* | Craythorne et al., (2022) | anonymous bullying too |  |  |
| *that I look like adog… saying that I’m ugly* | Craythorne et al., (2022) | appearance comparison in bullying |  |  |
| *I felt hurt that people wouldactually say it… but at the same time I was like ‘yeah you’reright’… ‘yeah I know I’m that – I just wish I wasn’t’* | Craythorne et al., (2022) | would believe the bullying content as true |  |  |
| *– I just wish I wasn’t’ (* | Craythorne et al., (2022) | internatlise content of bullying despite not liking it |  |  |
| *When I was at school, aged approximately seven,* | Craythorne et al., (2022) | bullying starting at school age |  |  |
| *I was sittingat a table with another boy… and two girls… one of the girlssaid something like* | Craythorne et al., (2022) | person able to recount and recall the bullying event vividly, as if iamgining |  |  |
| *I do remember in detail* | Craythorne et al., (2022) | vivid memory of the bullying |  |  |
| *that he found mysister attractive and I tried (very poorly on reflection) to flirtwith him by stating that ‘I am always told that we both lookalike so that must mean that I am attractive to [sic]?’…* | Craythorne et al., (2022) | from someone found attractive. appearance comparison to sister |  |  |
| *I distinctly remember the pause, the awkward laugh and thecomment ‘No, not really’… it is vivid in my mind during andafter the phone call I was staring in the mirror* | Craythorne et al., (2022) | use of wors vivid and distinctly remember, about the impactful memory |  |  |
| *remember feeling quite um crushed and almost erm takenaback really a bit in shock… it felt like the bottom of mystomach had fallen out* | Craythorne et al., (2022) | vivid emotional imagery of trauma |  |  |
| *During the period between the age of 12-16 I remember beingrejected by a few boys and I do think that was a contributingfactor.* | Craythorne et al., (2022) | rejection from people they feel are attractive |  |  |
| *Overall, I was heavily comparing myself to others andidolising girls who were slim and pretty.* | Craythorne et al., (2022) | self criticism and comparison to others perceived as more attractive |  |  |
| *My dad would get short tempered with me and tell me off,* | Craythorne et al., (2022) | parent short tempered |  |  |
| *I just have a sense ofnot feeling good enough in his eyes so whatever was saidcreated that belief in me* | Craythorne et al., (2022) | sense of ‘not good enough’ from non-aesthetic history, ie being seen as unimportant by father |  |  |
| *He once hit me (slapped across myface leaving a hand print) because I did something wrong andI remember the moment clearly, although I have no idea whatI did wrong* | Craythorne et al., (2022) | remember the bad thing but not the cause of it |  |  |
| *I also got told repeatedly when I was naughty orannoying that 'I was so much like my auntie* | Craythorne et al., (2022) | received a lot of critical remarks, not appearnce related, growing up |  |  |
| *She [Rohit’s mother] had an abusive nature, was harshlycritical and discouraging towards me. She would keeppointing out mistakes and negatives in me and everythingI did.* | Craythorne et al., (2022) | critical parent, non aethstic reasons |  |  |
| *I do notremember being appreciated or encouraged for anything byher.* | Craythorne et al., (2022) | no memories of being appreciated by caregiver |  |  |
| *I remember getting shamed often, in comparison to otherkids of my age, my cousins and friends* | Craythorne et al., (2022) | negative comparison to other sof same age |  |  |
| *made, even simple things like buyingthe wrong vegetables from the shop* | Craythorne et al., (2022) | critiqued for even simple thing |  |  |
| *vulnerable to developing a poor relationship with hisown sense of self during his formative years* | Craythorne et al., (2022) | vulnerability to poor sense of self and relatinoship with self |  |  |
| *I think I found an article about it [BDD] once and it soundedlike me or something like that happened I’m not sure* | Craythorne et al., (2022) | saw online content about bdd and related with it |  |  |
| *Of the 18 BDD patients who reported sponta-neous images/impressions, 15 88%) reported thatthey were associated with a particular stressfulmemory.* | Cooper & Osman, (2007) | almost all bdd, 88%, said their imagery was associated with a particular stressful memory |  |  |
| *memory was 11.50* | Cooper & Osman, (2007) | age they were at time of memory associated with imagery was 11 and a half |  |  |
| *Typical themes included:being teased and bullied at school e.g* | Cooper & Osman, (2007) | associated memories are around being bullied/teased at school |  |  |
| *I was 10years old and never got on with this boy in school* | Cooper & Osman, (2007) | person describes imagery memory being from age 10, about another child calling them ugly |  |  |
| *why he didn't likeme and he said `it's because you're ugly'.''* | Cooper & Osman, (2007) | person remembers someone saying they didn’t like them because they were ugly, at age 10 |  |  |
| *about appearance changesduring adolescence e.g.* | Cooper & Osman, (2007) | trauma associated to body iamge led to appearnce related self-consciousness during adolescent appearance changes |  |  |
| *My whole face and body seemed outof proportion* | Cooper & Osman, (2007) | memory, person remembers feeling different to others due to being tall, “out of proportion” |  |  |
| *all the early memories could be placedin one or both) of these two categories.* | Cooper & Osman, (2007) | study suggests all early memories are either about bullying in childhood, or self-consciousness about changes in appearance in adolescence |  |  |
| *Of the 15 control participants who reportedspontaneous images/impressions, only 2 13%)reported these to be closely linked to a particularmemory* | Cooper & Osman, (2007) | only 13% of controls without bdd said their imagery was related to a specific memory |  |  |
| *The median age in which the memoryoccurred was 13.00* | Cooper & Osman, (2007) | age of associated memory slightly higher for controls, at age 13 |  |  |
| *One patient did not wish to discusstheir memory.* | Cooper & Osman, (2007) | for one person, the associated bdd memory was so intense they declined to describe it in the study |  |  |
| *A total of 11 BDD participants 61%) and 2control participants 11%) reported early mem-ories associated with thoughts. T* | Cooper & Osman, (2007) | 61% of bdd vs 11% of controls had automatic thoughts (not imagery) associated with their early memories |  |  |
| *Most participants who brought in photographs of themselves asyoung children* | Silver & Reavey (2010) | participants able to use past phtographs of themselves to create imagery and empotional tone of self compared to past self |  |  |
| *young children constructed the younger self in an exceptionallypositive way and remembered childhood as a happy time* | Silver & Reavey (2010) | almost all people who related their childhood said they were more positive and happy compared to now |  |  |
| *However, the use of spotcream and its extreme effects on her skin seem to change her self-perception in a very dramatic manner* | Silver & Reavey (2010) | particpants could vividly describe the emotional relief brought on by early experiences of hiding, eg using spot cream or make up |  |  |
| *from human to monster (from typical teenager to outrageous ogre),which serves to distance herself from someone who is ‘normal’ andthus emphasises her anxieties and perceived defects.* | Silver & Reavey (2010) | suggested that the distortion people do in their appearnce imagery related to distancing perception of self from normality to their perceived defects generally as a person |  |  |
| *not really being… having theresources to deal with it – it wasn’t like – you know – any-itwasn’t- I don’t recall it being anything specific* | Craythorne et al., (2022) | vulnerability to bdd worsened by lack of resources to mange it at onset |  |  |
| *The mean age ofonset of BDD was 14.50 years SD = 7.68)* | Cooper & Osman, (2007) | bdd onset in adolesence |  |  |
